# Restriction associated DNA-genotyping at multiple spatial scales in *Arabidopsis lyrata* reveals signatures of pathogen-mediated selection

**DOI:** 10.1186/s12864-018-4806-7

**Published:** 2018-06-27

**Authors:** James Buckley, Eric B. Holub, Marcus A. Koch, Philippine Vergeer, Barbara K. Mable

**Affiliations:** 1 Institute of Biodiversity, Animal Health and Comparative Medicine, College of Medical, Veterinary and Life Sciences, University of Glasgow, Glasgow, G12 8QQ UK; 2 Adaptation to a Changing Environment, Institute of Integrative Biology, ETH Zürich, CH-8092 Zürich, Switzerland; 3 School of Life Sciences, Warwick Crop Centre, University of Warwick, Wellesbourne, CV35 9EF UK; 4 Centre for Organismal Studies (COS) Heidelberg, Biodiversity and Plant Systematics, Heidelberg University, D69120 Heidelberg, Germany; 5 Plant Ecology and Nature Conservation Group, Wageningen University, P.O.Box 47, 6700 AA Wageningen, The Netherlands

**Keywords:** Balancing selection, *Arabidopsis lyrata*, Genome scan, RAD-seq, Polymorphism, Mating system, Pathogens, *R-*genes, Disease resistance

## Abstract

**Background:**

Genome scans based on outlier analyses have revolutionized detection of genes involved in adaptive processes, but reports of some forms of selection, such as balancing selection, are still limited. It is unclear whether high throughput genotyping approaches for identification of single nucleotide polymorphisms have sufficient power to detect modes of selection expected to result in reduced genetic differentiation among populations. In this study, we used *Arabidopsis lyrata* to investigate whether signatures of balancing selection can be detected based on genomic smoothing of Restriction Associated DNA sequencing (RAD-seq) data. We compared how different sampling approaches (both within and between subspecies) and different background levels of polymorphism (inbreeding or outcrossing populations) affected the ability to detect genomic regions showing key signatures of balancing selection, specifically elevated polymorphism, reduced differentiation and shifts towards intermediate allele frequencies. We then tested whether candidate genes associated with disease resistance (*R*-gene analogs) were detected more frequently in these regions compared to other regions of the genome.

**Results:**

We found that genomic regions showing elevated polymorphism contained a significantly higher density of *R*-gene analogs predicted to be under pathogen-mediated selection than regions of non-elevated polymorphism, and that many of these also showed evidence for an intermediate site-frequency spectrum based on Tajima’s D. However, we found few genomic regions that showed both elevated polymorphism and reduced F_ST_ among populations, despite strong background levels of genetic differentiation among populations. This suggests either insufficient power to detect the reduced population structure predicted for genes under balancing selection using sparsely distributed RAD markers, or that other forms of diversifying selection are more common for the *R*-gene analogs tested.

**Conclusions:**

Genome scans based on a small number of individuals sampled from a wide range of populations were sufficient to confirm the relative scarcity of signatures of balancing selection across the genome, but also identified new potential disease resistance candidates within genomic regions showing signatures of balancing selection that would be strong candidates for further sequencing efforts.

**Electronic supplementary material:**

The online version of this article (10.1186/s12864-018-4806-7) contains supplementary material, which is available to authorized users.

## Background

High throughput sequencing and associated genotyping methods have revolutionized population genomic studies of population structure and selection and been applied to a wide range of species [1]. Such approaches have proved particularly useful for resolving phylogeographic structure in non-model taxa [2–4], but also for detecting signatures of selection across the genome and therefore identifying novel candidate loci being influenced by selection [1, 4–9]. Nevertheless, these different aims often greatly differ in their strategy for sampling populations and individuals of a species, with phylogeographic studies often sampling many populations to maximise range-wide variation and studies aimed at detecting signatures of selection sampling more extensively within a smaller number of populations. There is also a bias towards detecting signatures of divergent, positive selection, rather than other forms of selection [5, 10, 11], where extensive within population sampling is thought to be critical [12].

Evidence for balancing selection is thus more limited than directional selection and mostly restricted to studies involving whole genome or gene sequencing in a small set of organisms, including humans and pathogenic organisms [13–15], and more recently species of *Arabidopsis* [16] and *Drosophila* [17]. An important question is therefore whether this scarcity of evidence is due to balancing selection being limited in its role in shaping genome-wide patterns of diversity (e.g. [13]), or due to the challenges of identifying signatures of balancing selection as opposed to other types of selection [10].

Balancing selection maintains adaptive genetic variation among and within populations through different mechanisms: frequency-dependent selection, heterozygote advantage and fluctuating selection in time and space [18–20]. Spatial variation in selection can result in variation being reduced locally, but maintained at a broader geographic scale in different geographic regions or environments [21]. Balancing selection is generally characterized by an increased coalescence time compared to neutral expectations, leading to an excess of polymorphism in an extended genomic region linked to the selected variant, an excess of intermediate frequency alleles, increased heterozygosity, and reduced differentiation among populations [22, 23]. Several of these signatures of balancing selection, in particular elevated polymorphism or reduced differentiation, could be easier to detect by sampling few individuals from many diverged populations, rather than extensively within a few populations (e.g. [24]).

Testing for increased polymorphism or extended linkage disequilibrium (LD) is a useful starting point for detecting balancing selection, but such patterns alone are potentially confounded by similar signatures expected under diversifying selection or selective sweeps, respectively, as well as different rates of recombination across the genome [22]. Combining these signatures with additional population genetic statistics, such as reduced genetic differentiation (F_ST_) relative to neutral expectations or shifts in the site frequency spectra towards intermediate frequency alleles (positive Tajima’s D values [25]), can provide more robust support for balancing selection [4, 7, 26]. However, many of these additional signatures of balancing selection can also reflect non-selective processes. For example, elevated Tajima’s D values can be produced by a demographic bottleneck [27] and high background levels of polymorphism can make it more difficult to detect an excess of genetic diversity or heterozgyosity at a locus under balancing selection, particularly in highly outcrossing populations, where variation in rates of recombination across the genome might also be high [22]. It is therefore important to be able to account for demographic history when interpreting patterns of selection.

Most studies so far have used whole gene sequences or whole genome re-sequencing to detect genomic signatures of balancing selection, but genotyping by sequencing data such as Restriction Associated DNA (RAD) sequencing could also be used to detect such signatures [28]. The short length of RAD markers (around 100 bp) and uneven spacing across genomes limits the use of traditional LD-based approaches (e.g. [29]), and the relatively low density of markers may limit the ability to detect SNPs linked to candidate genes under selection ([30], but see [9]). However, when reference genomes are available and higher numbers of loci used, sliding window-based approaches can be used to identify outlying genomic regions (groups of neighbouring loci) showing unusually high or low levels of polymorphism, genetic differentiation and allele frequencies within populations (e.g. [4, 7]). Identifying genomic regions showing multiple signatures of balancing selection provides a general test for how common this form of balancing selection is across the genome, but would also highlight candidate loci in these regions likely to be influenced by balancing selection.

The potential complexity in patterns of polymorphism at loci under balancing selection means that special attention should also be given to filtering and assembly of high throughput sequencing data. As many genes under this type of selection are members of large gene families characterized by copy number variation (e.g. [10, 31, 32]), filtering SNPs to minimise the amount of missing data or remove potential paralogs could inadvertently exclude the strongest candidates [33]. Furthermore, an excess of predicted polymorphism might result from alleles being incorrectly assembled or aligned to reference genomes. Thus, genome scans for balancing selection might also require both different levels of sampling and different filtering decisions than tests for other types of selection.

Genes involved in ligand-mediated recognition processes in animals and plants, such as immune genes, plant disease resistance genes (*R*-genes), and genes controlling self-incompatibility are often cited as classic examples of balancing selection [20, 34]. In plants, large families of *R*-gene analogs (RGAs) contain particular motifs based on conserved structural features [35–37]. A leucine rich repeat (LRR) motif is a shared domain in the three main plant RGA families, including two classes of cell surface receptors (receptor-like kinases, RLKs; and receptor-like proteins, RLPs) and a class of cytoplasmic receptors (nucleotide binding site-leucine rich repeat proteins, NLRs). RGAs therefore make a good test panel for investigating the power of genome scan approaches to detect balancing selection.

Whole genome sequencing of 20 wild *A. thaliana* accessions showed that the density of NLR encoding genes positively predicted levels of polymorphism across the genome [38]. Interestingly, sequencing of RGAs from a broad-scale sampling of *Arabidopsis thaliana* accessions has revealed genes showing clear signatures of balancing selection, but also others showing patterns more consistent with neutrality or directional selection [39–41]. As well as high levels of polymorphism, some plant NLR genes show presence and absence of functional alleles that confer resistance to specific pathogen genotypes, and many show copy number variation across related species [42]. By contrast, other RGA families, such as RLKs or RLPs, are not predicted to show such elevated polymorphism, as they are involved in more conserved interactions with PAMPs and MAMPs (Pathogen- and Microbe-associated molecular patterns) [35].

Given that most of our knowledge of RGAs is restricted to the highly selfing *A. thaliana*, outcrossing *Arabidopsis* relatives offer a great opportunity to explore the effects of differing ecology on such loci associated with adaptation [16, 43, 44]. *Arabidopsis lyrata* has an annotated reference genome [45] and shows a restricted ecological distribution, with strong population structuring both within North America (*A. lyrata* subsp*. lyrata,* in the following simplified as *A. l. lyrata*) and Europe (*A. lyrata* subsp. *petraea,* in the following simplified as *A. l. petraea*), as well as extensive differentiation between the subspecies [5, 46, 47]. *A. l. petraea* shows higher diversity than *A. l. lyrata,* with the highest diversity found in central European populations (Germany and Austria, thought to represent the centre of ancestral diversity in a glacial refugium; [46, 48, 49], a region that also shows extensive introgression with related *Arabidopsis* taxa [50]. A recent study based on whole genome re-sequencing confirmed a bottleneck in North America, and postglacial expansion from Central Europe into Scandinavia and the United Kingdom [5], with relatively deep divergence times among the European regions. Thus, there is substantial and well-characterised population structure to provide a framework for investigating patterns of selection. In previous work, targeted sequencing of two *R*-gene analogs detected weak evidence for balancing selection in *A. lyrata*, despite strong evidence in *A. thaliana,* but emphasised that tests for selection are sensitive to the relative divergence among populations included in the analysis, since a stronger signature was detected by comparing subspecies rather than within subspecies [51].

North American populations also show variation in mating system within a limited geographic area in the Great Lakes regions [52–55]. Selfing populations show substantially reduced genetic diversity and heterozygosity compared to outcrossing populations in the same geographic region, as well as evidence of independent post-glacial colonization [54] and a severe bottleneck evidenced by a reduced number of self-incompatibility alleles [56]. The low levels of background polymorphism observed in these selfing populations could make it easier to detect elevated diversity and heterozygosity at loci under balancing selection.

In this study, we used RAD-sequencing of *A. lyrata* samples from the two subspecies to: (1) compare the resolution of broad- and finer-scale patterns of phylogeographic structure and genetic variation within these subspecies with previous studies using microsatellite loci or single nucleotide polymorphism genotyping based on relatively few genes; (2) identify regions of the genome showing deviations from this neutral structure characteristic of balancing selection; and (3) test the ability of different sampling strategies to detect balancing selection using a test panel of RGAs. We focused our analyses on different subsets of samples from the two subspecies to identify genome-wide patterns of polymorphism and divergence. Specifically, we tested for: (a) high diversity and heterozygosity in otherwise low diversity genetic backgrounds, using selfing populations of *A. l. lyrata* from the North American Great Lakes; and (b) reduced population genetic structure and changes in site frequency spectra, using different sets of outcrossing populations sharing a recent postglacial demographic history. We also tested the sensitivity of conclusions to filtering strategies, and compared genome scans using both individual RAD loci and smoothed genomic regions based on sliding window analyses.

We show that genotyping just a few individuals per location at multiple spatial scales can both resolve patterns of phylogeographic structure, but also identify genomic regions showing signatures of balancing or diversifying selection that contain candidate disease resistance loci.

## Results

### Spatially arranged genetic structure, diversity and heterozygosity across multiple spatial scales in *A. lyrata*

**Fig. 1:**
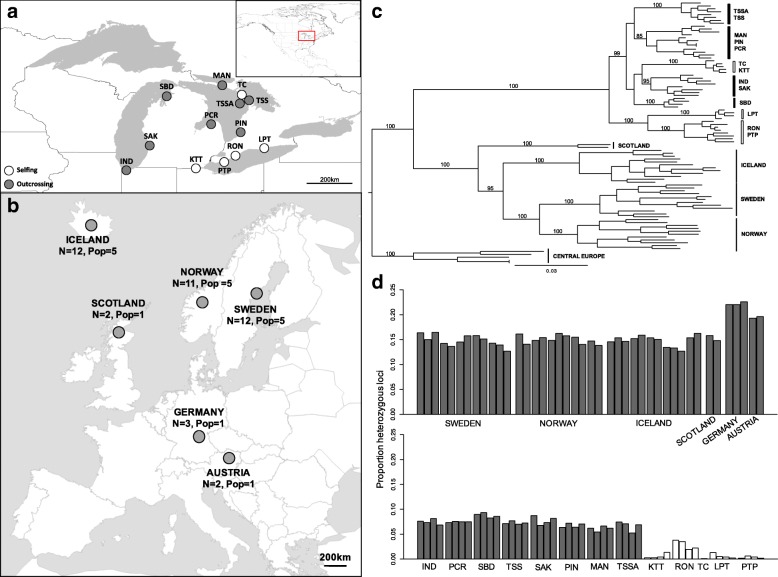
Summary of populations sampling, phylogenetic relationships and individual heterozygosity based on RAD loci. Maps of sampling locations for: (**a**) the North American Great Lakes, and (**b**) Northern and Central European populations. For the former, predominantly outcrossing populations are indicated by grey circles and highly selfing populations by white circles (*N* = 4 per population, except TC, *N* = 1). For Europe, the sample size per country and number of populations from which these samples originate are given on the figure. (**c**) RaXML tree based on 15,571 SNPs across 5936 RAD loci. Bootstrap support values greater than 70 are given for major branches denoting groups indicted on the right of the tree (countries for European samples and populations for North American samples); for the latter, clear bars indicate selfing populations and black bars outcrossing samples. Central Europe refers to samples from Germany and Austria. (**d**) Individual heterozygosity (proportion of heterozygous loci, H_o_) estimates using 6665 RAD loci present in all individuals (including non-polymorphic loci)

We genotyped 91 individuals across the subspecies of *A. lyrata* using RAD-seq (Fig. 1a, b; Additional file 1: Table S1a,b) at 5942 loci present in all individuals and confirmed phylogeographic patterns consistent with neutral expectations, both in terms of levels of polymorphism in different geographic regions and genetic relationships among populations. Despite there being on average more reads for European samples (Additional file 1: Figure S1a), a similar number of reads aligned uniquely to the reference sequence (Additional file 1: Figure S1b), and to multiple places in the genome (Additional file 1: Figure S1c) for European and North American samples. However, as expected, given that the reference genome sequence derives from a North American individual (the IND population; [45]), there were significantly lower numbers of unaligned reads for North American than European samples (Additional file 1: Figure S1d). Levels of polymorphism were lowest in North American selfing samples and highest in central European samples (Fig. 1d; Additional file 1: Table S2). Comparisons between observed (*H*_*o*_) and expected heterozygosity (*H*_*e*_) suggested an overall heterozygote deficit in all geographic regions, but this was strongest in the selfing samples, as might be expected with inbreeding (Additional file 1: Table S2; [54]).

A maximum likelihood tree (RaXML) based on 15,571 SNPs (from 5936 RAD loci) shared across all samples showed strong support for separation of the two subspecies, as well as central European populations from Northern European populations (Fig. 1c), consistent with previous studies [5, 46]. Within the European (*A. l. petraea*) clade, individuals clustered by country of origin, with 100% bootstrap support. The North American (*A. l. lyrata*) individuals were separated into two main clusters, with individuals from selfing populations allocated to both supported clades, consistent with previous observations of multiple independent evolutionary origins of selfing in this region [54]. Additionally, outcrossing populations formed three well-supported clusters (MAN, PCR, PIN vs IND, SAK, SBD vs TSS, TSSA; Fig. 1c), again broadly consistent with previously published STRUCTURE results [54].

**Fig. 2:**
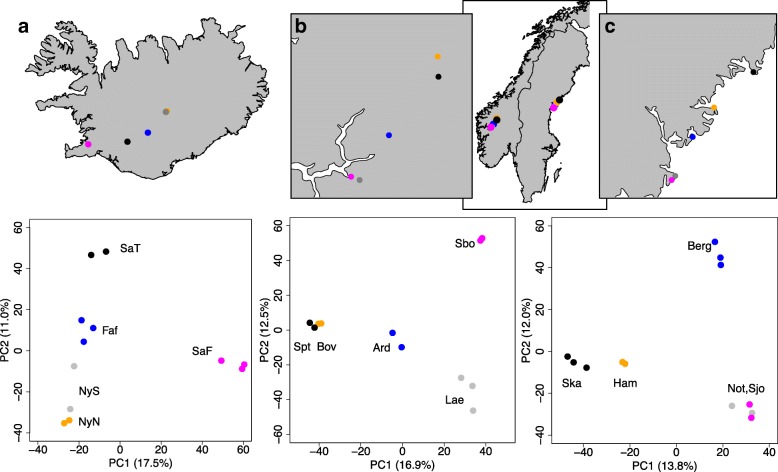
PCA genetic clustering of populations within the three Northern European countries. Genetic structure as described by principal components axis 1 and 2 for samples from 5 populations from each of: (**a**) Iceland (12 samples); (**b**) Norway (11 samples); and (**c**) Sweden (12 samples). Colours and site code names (see Additional file 1: Table S1) denote different sample populations within each country

Principal Components Analysis confirmed the separation of Central European (EU-C) and Northern European (EU-N) groups (Additional file 1: Figure S2a), as well as clustering by country of origin across Northern Europe (Additional file 1: Figure S2b). Similar broad-scale geographic genetic structure has been observed using neutral gene sequences [46] and more recently, whole genome resequencing [5]. Even with just two or three individuals per population the PCA revealed clear genetic differentiation among most populations within Scandinavian countries (Fig. 2a-c). Furthermore, we detected significant isolation-by-distance patterns across the five populations within Norway (F = 12.03, *p* = 0.050; Additional file 1: Figure S3a) and Iceland (F = 16.86, *p* = 0.018; Additional file 1: Figure S3c), and a non-significant, but positive relationship across populations within Sweden (F = 2.55, *p* = 0.1473; Additional file 1: Figure S3b).

**Fig. 3:**
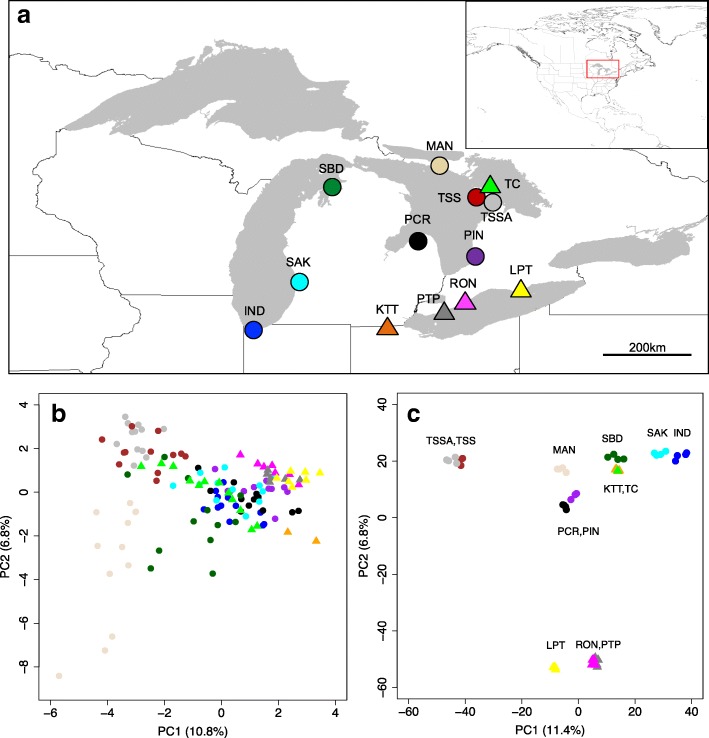
Comparison of genetic clustering around the Great Lakes using RAD loci and microsatellites. The geographic location (**a**) of North American Great Lakes populations and their genetic clustering with respect to the first two principal component axes, using: (**b**) eight microsatellite loci (from Foxe et al. 2010) and (**c**) 3337 polymorphic RAD loci. Colours distinguish samples from different collection sites, circles represent predominantly outcrossing and triangles highly selfing populations

For the North American samples, we observed much stronger population-level PCA clustering, than previously observed using twice as many individuals genotyped at eight microsatellite markers (Fig. 3). The clustering was consistent with geographic separation of populations on lake Michigan (SBD, SAK, IND) and Erie (LPT, RON, PTP), as well as two sets of populations located on Lake Huron (TSS/TSSA and PIN/PCR). A geographically intermediate population (MAN, located on Manitoulin Island) was also intermediate in the PCA. Two geographically distinct highly selfing populations (TC and KTT) clustered together, but we could only genotype a single individual from TC. The regional sub-structuring observed using PCA and the RaXML tree might explain the absence of a significant relationship between genetic and geographic distance for these North American outcrossing (NA-O) populations (F = 1.197389, *p* = 0.2701, Additional file 1: Figure S3d).

### Detecting signatures of balancing selection at multiple spatial scales

#### (A) Identifying loci showing elevated observed heterozygosity and diversity in a low variation background

Previous studies have used evidence of elevated diversity and observed heterozygosity in selfing or inbred populations to detect loci under balancing selection [22, 40, 57], so we predicted that genomic regions showing elevated polymorphism at RAD loci in the North American selfing (NA-S) populations might contain genes exhibiting allelic variation under balancing selection.

We first tested how varying the amount of missing data affected the density of SNPs and coverage per RAD locus, as well as summary statistics describing patterns of polymorphism and heterozygosity. Allowing for loci to be either present in all individuals (0% missing), or missing in up to 50% individuals (< 50% missing), as might be expected for highly polymorphic genes that do not consistently align to the genome due to divergence from the reference (e.g. [33, 56]), increased the number of RAD loci to 16,240 loci (2.7× higher; Additional file 1: Table S3a; Additional file 1: Figure S4). In both cases, the density of RAD reads was also reduced around the centromere of each chromosome, likely due to an increase in repetitive DNA and poor centromere assembly in the reference sequence [45]. Differences in the proportion of heterozygous loci among individuals were surprisingly consistent with or without missing data (Fig. 1d vs Additional file 1: Figure S5), suggesting that including more variable loci with missing data does not dramatically alter overall patterns of observed heterozygosity between individuals (Additional file 1: Table S2; Fig. 1d vs Additional file 1: Figure S5). However, including loci with missing data did slightly increase average polymorphism summary statistics (e.g. nucleotide diversity was 1.07–1.15-fold higher in each sample group when allowing for missing data; Additional file 1: Table S2), which suggests that RAD loci in more polymorphic regions of the genome were being included in the dataset. As a result of this and the higher density of RAD markers, we therefore used loci present in at least 50% individuals for analyses of other *A. lyrata* sample groups.

Using estimates of nucleotide diversity, expected heterozygosity and observed heterozygosity (proportion of heterozygous individuals) at individual RAD loci we then identified individual outlier RAD loci with the 1% highest levels of polymorphism in the North American selfing (NA-S) group (see Additional file 1 for details). Several loci were excluded from the single outlier analyses due to uncertainty about assembly (Additional file 1: Table S4a). However, as predicted, more candidate outlier loci were identified when allowing loci to be missing in individuals than when only using those loci present in all individuals (Additional file 1: Table S4a). A BLAST search of RAD outlier sequences to a database of *A. thaliana* coding sequences (TAIR10.29) revealed four RAD loci annotated as NLR genes when allowing for up to 50% missing data, but only two using 0% missing data (Additional file 1: Table S4a,b; details in Additional file 1: results).

**Fig. 4:**
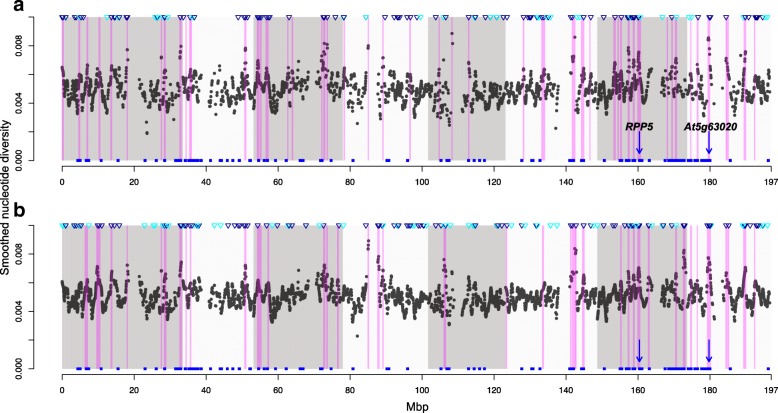
Genomic regions of elevated polymorphism and location of NLR genes across North American selfing populations. Smoothed nucleotide diversity plots based on (**a**) 4327 loci present in all, or (**b**) 7256 loci present in at least 50% of individuals. Regions of significantly elevated nucleotide and/or gene diversity (neighbouring windows, 450 kbp either side of a polymorphic locus; *p* < 0.05) are marked in purple, with the eight chromosomes shaded alternately in dark and light grey. The genomic locations of single outlier loci are marked along the top of the graph by downward pointing blue triangles (dark blue = single outlier based on nucleotide and gene diversity, or turquoise = based on observed heterozygosity). The location of 206 annotated NLR genes on the *A. lyrata* reference chromosomes 1–8 are denoted by blue squares, with two candidates (*RPP5* [95] and *At5g63020* [40]) marked by downward pointing blue arrows. These annotated candidates are present in high diversity regions for both sets of RAD loci and show a priori evidence for balancing selection in *A. thaliana* (Additional file 1: Table S6)

Genome-wide variation in nucleotide diversity and expected heterozygosity was then estimated in smoothed windows centred on polymorphic RAD loci using the programme *Stacks* [58], with bootstrapping used to test the significance of elevated windows of polymorphism relative to the genome-wide average. For NA-S samples the smoothed window analysis was based on 900Kbp windows, with 410 windows of significantly elevated diversity present in all individuals and 542 windows present in at least 50% individuals (Table 1a). Large window sizes were necessary due to the low number of polymorphic RAD loci across NA-S samples (4327 loci present in all individuals, 7256 loci with up to 50% missing data), which limits the fine-scale resolution of this approach. Nevertheless, we predicted that Nucleotide Binding-Leucine Rich Repeat (NLR) loci (listed in Additional file 1: Table S5) would be at higher frequency in windows of significantly elevated diversity than in other smoothed windows, because they are expected to be under pathogen-mediated balancing or diversifying selection. By randomising the label of smoothed windows as ‘high diversity’ or ‘not high diversity’ we produced a null distribution of the difference in the number of NLR genes in each set of windows. We found significantly higher numbers of NLR loci in high diversity windows relative to all other windows (*p* < 0.0001; Table 2a; Additional file 1: Figure S6a,b), irrespective of the filtering criteria used and despite the large window size encompassing many *A. lyrata* genes (Table 2a). For example, the start of chromosome 2, middle and end of chromosomes 7, and start of chromosome 8 showed a high density of significantly elevated diversity windows and high NLR gene density (Fig. 4a, b). By contrast, the density of another set of annotated RGAs, the LRR-RLK genes for which elevated diversity was not predicted, was not higher in windows of elevated diversity (*p* = 0.221; Table 2b, Additional file 1: Figure S7a,b).

**Table 1:** High diversity genomic regions and associated disease resistance genes

	Smoothing parameters		Number windows showing significantly (*p* < 0.05) elevated polymorphism					
a)								
Group^a^	Proportion missing data	Window size parameter, σ (Kbp)	Total number of windows	*H*_*e*_	*π*	*H*_*e*_ *and π*	*Total*	Proportion showing significantly elevated polymorphism
NA-S	0	150	4327	151	165	94	410	0.0948
NA-S	< 50%	150	7256	163	225	154	542	0.0747
EU-N	< 50%	60	16,990	393	190	491	1074	0.0632
NA-O	< 50%	60	14,572	555	307	966	1828	0.1254
ALL-O	< 50%	50	23,465	452	478	917	1847	0.0787
b)								
Group^a^	Proportion missing data	Number regions of high diversity	Size of regions (Mbp)	Number genes in regions		Number NLR genes in region		Proportion of all NLR genes
NA-S	0	50	24.351600	4355		52		0.250
NA-S	< 50%	43	22.346912	3710		49		0.238
EU-N	< 50%	128	22.903806	3647		64		0.311
NA-O	< 50%	156	31.039876	5575		90		0.437
ALL-O	< 50%	179	28.702652	4406		75		0.364

(a) the number of smoothed high diversity windows with significantly elevated *H*_*e*_ (expected heterozygosity), *π* (nucleotide diversity) or both for different sample groups; and (b) the number of candidate NLR loci (Nucleotide binding site- Leucine rich repeat genes described in test panel in Additional file 1: Table S5) in genomic regions of significantly elevated diversity. Genomic regions are based on combining smoothed windows of significantly elevated diversity that overlap along a chromosome

^a^NA-S = N. American selfing, EU-N = Northern European (Sweden, Iceland and Norway), NA-O = N. American outcrossing, ALL-O = all outcrossing samples i.e. without NA-S samples

**Table 2:** Elevated density of disease resistance candidates in areas of high genomic polymorphism

a)		Mean number NLR genes		
Group^a^	Proportion missing data	All high diversity windows	All other windows	Significance of difference
NA-S	0	0.615	0.299	***
NA-S	< 50%	0.738	0.332	***
EU-N	< 50%	0.331	0.111	***
NA-O	< 50%	0.402	0.102	***
ALL-O	< 50%	0.332	0.080	***
b)		Mean number LRR-RLK genes		
Group^a^	Proportion missing data	All high diversity windows	All other windows	Significance of difference
NA-S	0	0.390	0.486	*
NA-S	< 50%	0.443	0.491	ns
EU-N	< 50%	0.156	0.206	*
NA-O	< 50%	0.172	0.194	ns
ALL-O	< 50%	0.140	0.171	*

(a) The average number of NLR genes and (b) LRR-RLK genes in significantly elevated diversity windows and all other windows are given, along with the significance of the difference in average number of genes per window based on a randomisation test. These averages are based on numbers of genes present in individual smoothed windows defined by *Stacks* and significance of the difference is denoted as *** *p* < 0.0001, ** *p* < 0.001, * *p* < 0.01, ns = *p* > 0.05

^a^ NA-S = N. American selfing, EU-N = Northern European (Sweden, Iceland and Norway), NA-O = N. American outcrossing, ALL-O = all outcrossing samples i.e. without NA-S samples

By merging adjacent smoothed windows of significantly elevated diversity found using loci present in all individuals, we identified 50 genomic regions of significantly elevated diversity covering 24.35Mbp (12% of chromosomes 1–8; Table 1b) and containing 4355 *A. lyrata* genes (14% of all genes on chromosomes 1–8). Of these, 52 were annotated as NLR encoding genes present in our test panel (Additional file 1: Table S5) and eight exhibited variation attributed to balancing selection in other studies (summarized in Additional file 1: Table S6). Similarly, using loci with < 50% missing data, the windows of significantly elevated diversity merged into 43 genomic regions of significantly elevated diversity covering 22.35 Mbp (11% of total genome; Table 1a, b) and containing 3710 genes (12% of total genes). Of these, 49 were annotated as NLR encoding genes and included three that exhibited variation attributed to balancing selection in *A. thaliana* or *Capsella* species (Additional file 1: Table S6). In contrast to the individual RAD outlier analysis, these data suggest that varying the amount of missing data does not dramatically alter the number of disease resistance candidates detected.

However, NLR encoding genes present in genomic regions of elevated diversity did not fully overlap when allowing for 0% or < 50% missing data. Of the 62 NLRs identified in total across both filtering strategies, 62.9% (39 loci) were shared between the analyses with 0% or < 50% missing data. Of these shared candidates, three genes were annotated as two *A. thaliana* NLRs, specifically *At4g16950* (RPS2: [59]) and *At5g63020* (no known function), both with prior evidence for balancing selection (Additional file 1: Additional file 1: Table S6; genomic position indicated in Fig. 4). Interestingly, three of the four candidate genes identified by the single outlier analyses in the NA-S comparison were also found in smoothed genomic regions of elevated diversity (Additional file 1: Table S4b), in addition to many new candidate NLRs. We therefore adopted only the smoothing approach for the remaining analyses.

#### (B) Identifying loci showing elevated diversity, reduced genetic differentiation and intermediate allele frequencies in outcrossing populations

Balancing selection may also leave a signature of reduced genetic differentiation among populations, as well as shifts in the allele frequency spectrum towards intermediate allele frequencies [22, 23]. These can be observed, respectively, by lower estimates of genetic differentiation, F_ST_, among populations relative to the genome-wide average, and elevated Tajima’s D relative to the genome-wide average. We used 35 outcrossing individuals of *A. lyrata* from 15 populations (originating from three countries) in Northern Europe (EU-N) and 32 individuals from eight outcrossing populations around the North American Great Lakes (NA-O), for which genetic structure among populations within each region is strong (Fig. 3, Additional file 1: Figure S2b). Specifically, we identified all genomic windows showing both significantly elevated diversity and either signatures of reduced F_ST_ or positive Tajima’s D (Additional file 1: Table S7) using 16,990 polymorphic loci present in at least 50% of EU-N samples and 14,572 polymorphic loci present in at least 50% of NA-O samples. The number of polymorphic RAD loci is notably lower than the 64,056 predicted using the *Pst1* enzyme, partly because one third of loci are not being assembled in *Stacks* (e.g. reads stochastically absent from a sample, or not aligned singly to the genome), but mostly due to the requirements for loci to be present in no fewer than 50% individuals in a sample group (see methods for details). Using loci present in fewer than 50% of individuals in a sampling group increased the density of loci, but also resulted in low sample sizes to estimate diversity and differentiation at individual loci, which we wanted to avoid.

First, we identified smoothed windows of significantly elevated diversity (H_e_ and/or π) for EU-N and NA-O samples separately using 300Kbp windows centred on polymorphic RAD loci. This is a broad window size, but given that there were polymorphic RAD loci on average every 11.595Kbp for NA-O samples and 13.519Kbp for EU-N samples, and *Stacks* estimates average window polymorphism with reduced weight on RAD loci further from the centre of the window, we are confident that the effective resolution of our windows was more localised than the total window size suggests. Generally, there was some overlap in the location of high diversity windows in the EU-N and NA-O samples (Additional file 1: Figure S8a vs S8b), although the proportion of windows showing significantly elevated diversity was higher (12.5%) for the NA-O samples than for the EU-N samples (6.3%, Table 1a), likely due to the higher overall diversity across the EU-N samples.

**Fig. 5:**
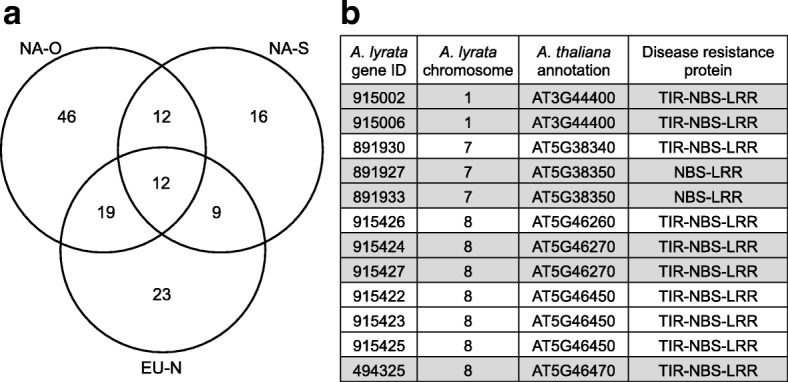
Disease resistance loci in genomic regions of significantly elevated diversity shared between sample groups. **a** Venn diagram illustrating the number of candidate *A. lyrata* genes unique to and shared between different groups (NA-O, NA-S and EU-N sample groups). This analysis is based on RAD loci with up to 50% individuals missing within each group; (**b**) a list of the 12 annotated NLR genes that were shared between all groups. Note that some *A. lyrata* genes have the same TAIR annotations for *A. thaliana* (see highlighted sections)

As with the selfing samples, NLR-encoding genes were found at significantly higher densities in smoothed genomic windows of significantly elevated diversity relative to all other windows (randomisation test: *p* < 0.0001 for both EU-N and NA-O; Table 2b; Additional file 1: Figure S6c,d). By contrast, LRR-RLK genes were either at higher frequency in smoothed windows of non-elevated diversity or showed no significant difference (EU-N: *p* = 0.004, NA-O: *p* = 0.067, Table 2b, Additional file 1: Figure S7c,d). When individual windows were merged into larger genomic regions of elevated diversity, there were on average 2.8 and 2.9 NLRs per Mbp in regions of significantly elevated diversity for EU-N and NA-O samples respectively (compared to a genome-wide average of 1.0 NLR per Mbp). A total of 31 NLR genes were found to be in high diversity genomic regions in both sample groups (Fig. 5; Additional file 1: Figure S8), including eight genes showing evidence for balancing selection in other studies (Additional file 1: Table S6). However, just 12 NLRs (9.6% of all NLRs identified; Fig. 5a, b) were found to be present in high diversity genomic regions in all three sample groups: EU-N, NA-O and NA-S. Interestingly, none of these loci have yet been described as showing signatures of balancing selection in other species.

**Fig. 6:**
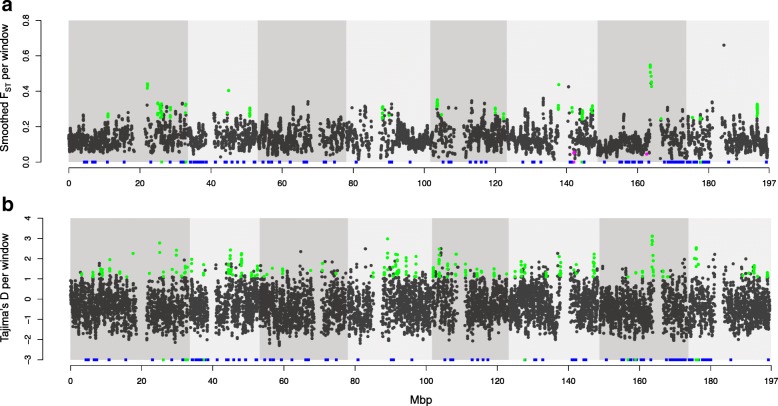
Patterns of genetic differentiation and Tajima’s D in sliding genomic windows across the genome of NA-O samples*.* Smoothed genetic differentiation (F_ST_) and sliding window analysis of Tajima’s D across the genome of the NA-O samples. **a** Smoothed genetic differentiation among three NA-O genetic structure groups estimated using loci present in at least 50% of individuals within each group. (b) 120Kbp sliding window analysis of Tajima’s D, across the genome of all NA-O samples (*N* = 28). Windows showing elevated (green) or reduced (magenta) F_ST_ in a region of elevated diversity are indicated in (**a**) and (**c**), with regions of elevated Tajima’s D in regions of significantly elevated diversity (green) indicated in (**b**) and (**d**). The location of 206 annotated NLR genes on the *A. lyrata* reference chromosomes 1–8 are denoted by blue squares, with those in the windows overlapping with regions of elevated diversity highlighted in green or magenta. A similar set of plots are given for all EU-N samples in Additional file 1: Figure S10

To attempt to distinguish balancing from other types of selection, we then identified smoothed windows showing reduced F_ST_ between geographic regions (EU-N or NA-O) or positive Tajima’s D (suggesting shifts towards intermediate allele frequencies) within geographic regions that overlapped with smoothed windows of significantly elevated diversity in one or both sample groups. For the EU-N group, just five smoothed windows of significantly elevated diversity (out of 1074 in total) showed F_ST_ values in the bottom 5% of windows (Additional file 1: Figure S10a). These windows merged into three genomic regions that together were 0.36 Mbp in length and contained 26 genes, but no NLR genes. For the NA-O group only 11 windows of significantly elevated diversity (out of 1828 in total) had F_ST_ estimates in the bottom 5% of all windows (Fig. 6a). These windows merged into two genomic regions that were 0.20Mbp and 0.12Mbp in length and respectively contained 19 and 17 genes, of which two were NLRs (both on Chromosome 6) involved in interactions with oomycete pathogens in *A. thaliana: At2g14080 (RPP28;* Sepahvand and Holub, unpublished) and *At3g44630 (RPP1* cluster gene; [39]).

Interestingly, a high proportion of windows of significantly elevated diversity overlapped with windows of high genetic differentiation (in the top 5% F_ST_ values) across both EU-N countries (Additional file 1: Figure S10a) and NA-O genetic clusters (Fig. 6a). For EU-N samples, there were 137 overlapping windows (3.804Mbp in length) containing 601 genes, of which nine were NLRs (summarised in Additional file 1: Table S7), and six showed signatures of balancing selection in previous studies (Additional file 1: Table S6). Two loci were annotated as *At1g63730* (uncharacterised resistance function), another two were annotated as *At1g63870* (*RLM-B* homolog involved in fungal resistance; Staal et al. 2006), and the final two were both annotated as *At5g43470* (*RPP8,* involved in oomycete resistance; McDowell et al. 1998). For NA-O samples, there were 182 overlapping windows of high diversity and F_ST_ (5.17Mbp in length) containing 791 genes, of which five were annotated as NLR encoding genes (Additional file 1: Table S7). One locus was annotated as *At1g50180* (uncharacterised resistance function), two as *At3g44400* (*RPP1* cluster; [39]) and a final locus as *At4g11340* (uncharacterised resistance function).

Finally, we identified smoothed windows showing significantly elevated diversity that overlapped with 120 Kbp sliding windows showing elevated Tajima’s D. To minimise the confounding effects of demographic history across countries, we identified the windows with the 5% highest values of Tajima’s D for each Northern European country separately (Norway, Sweden and Iceland) and for all NA-O samples combined. The average value across all SNPs for Northern European countries was consistently positive (Norway D = 0.547, Sweden D = 0.481, Iceland D = 0.722; Additional file 1: Figure S9b-d), but for NA-O was negative (− 0.395; Additional file 1: Figure S9a).

Interestingly, approximately 25% of the smoothed windows of elevated Tajima’s D also overlapped with smoothed windows of significantly elevated diversity in each Northern European country (Norway = 25.3%, Sweden = 29.3%, Iceland = 23.8%; Table 3; Additional file 1: Figure S10b,c,d). NLR encoding genes were identified in these windows, ranging from five in Sweden to 12 in Norway (Table 3). Most of the NLR candidates were unique to one country, with just four NLRs identified in overlapping windows in more than one country (Table 3). One *A. lyrata* NLR*-*gene on chromosome 7 (annotated as *At5g38850*) was identified in all three Scandinavian (EU-N) countries, although to our knowledge no functional resistance allele has been described in *A. thaliana* (Additional file 1: Table S7).

**Table 3:** Numbers of overlapping windows of elevated diversity and D for different geographic regions

	EU-N^a^			NA-O^b^
	Norway (*N* = 11)	Sweden (*N* = 12)	Iceland (N = 12)	(*N* = 32)
Total windows	13,679	13,650	13,607	14,278
High Tajima’s D and high diversity overlap	173	200	162	415
Number *A. lyrata* genes in overlap	904	1060	883	2321
Number NLR genes in overlap	12	5	8	33
Number country-specific NLR genes	9	2	5	–

The total number of windows in which Tajima’s D could be estimated (i.e. contained a SNP) is given for each of the Northern European countries (Norway, Sweden, Iceland) and all North American outcrossing samples. The overlap between windows with elevated Tajima’s D (top 5% of all windows) and significantly elevated diversity is also given, as well as the total number of predicted *A. lyrata* genes and genes annotated as NLR loci in the overlapping high Tajima’s D and high diversity windows. For the Northern European samples, the number of NLR genes identified in overlapping genomic regions in just one country

^a^ Northern European countries, ^b^ N. American outcrossing

For the NA-O sample set, 58.1% of windows of elevated Tajima’s D overlapped with windows of significantly elevated diversity (Table 3; Fig. 6b), and a greater number of known NLRs were identified in these overlapping regions compared to the Scandinavian samples (33 putative NLR encoding genes in total). Of particular interest were three NLR*-*genes (two annotated as *At1g56540*, and another annotated as *At1g59780*) that also show elevated polymorphism and high Tajima’s D in *A. thaliana* (Additional file 1: Table S6)*.* Both are located in gene clusters associated with resistance to oomycete pathogens in *A. thaliana* [40, 60].

To potentially increase power for detecting signatures of reduced genetic differentiation we also assessed evidence for elevated diversity windows across all outcrossing samples (NA-O, EU-N and including central European and Scottish samples). In total 1847 windows showed significantly elevated H_e_ and/or π, which is higher than that observed for the individual sample groups (NA-O, EU-N and NA-S). NLRs were again significantly overrepresented in windows of elevated diversity (*p* < 0.0001; Table 1b; Additional file 1: Figure S6e), whereas LRR-RLK genes were at slightly higher frequency in windows of non-elevated diversity (*p* = 0.01, Table 2b, Additional file 1: Figure S7e). These windows merged into 179 genomic regions (28.7 Mbp in length) of elevated diversity distributed across the genome (Additional file 1: Figure S11a). Of the 4406 genes in these regions, 75 were NLR*-*encoding genes with 10 loci annotated as *A. thaliana* resistance genes that have been described as under selection in studies of either *A. thaliana* or *Capsella* species (Additional file 1: Table S6), including genes annotated as *RPP1, RPP13, RPP7* and a paralog of *WRR4* (Additional file 1: Table S6).

On average, smoothed F_ST_ between the two subspecies was 0.278 (Additional file 1: Figure S11b), which should be a sufficiently high baseline for detecting windows of reduced genetic differentiation that overlap with windows of elevated diversity. However, only one elevated diversity window on chromosome 7 also displayed a smoothed F_ST_ value in the bottom 5% of all windows (highlighted in pink in Additional file 1: Figure S11b). Interestingly, this window contained two NLR encoding genes annotated as *At5g40090* and *At5g40100*, although to our knowledge there are no prior reports of these loci being involved in interactions with pathogens (Additional file 1: Table S7). As observed for the EU-N and NA-O groups, the overlap between windows of significantly elevated diversity and those with F_ST_ values in the top 5% of all windows was much higher (Additional file 1: Figure S11b). Of 494 overlapping windows (11.66 Mbp in length) there were 12 NLR*-*encoding genes, most of which represent NLR genes with uncharacterised functions in *A. thaliana* (summarised in Additional file 1: Table S7).

## Discussion

In this study we demonstrate that high throughput genotyping of a small number of individuals across a large number of populations can identify signatures of different forms of selection. Specifically, we identified genome-wide signatures of balancing and diversifying selection within and between two subspecies of *A. lyrata*, and then focused on whether annotated disease resistance genes predicted to be under pathogen-mediated selection were over-represented in genomic regions showing elevated polymorphism. Our results reveal three key findings: (1) we confirmed previously observed patterns of genetic diversity and genetic structure across the subspecies of *A. lyrata*, but also revealed finer-scale spatial genetic structure within countries using only several individuals per population; (2) genomic regions showing elevated polymorphism contained a significantly higher number of disease resistance genes than other genomic windows; and (3) relatively few genomic regions showed both elevated diversity and reduced genetic differentiation among sample groups, a key signature distinguishing balancing from other forms of selection.

### Spatially arranged genetic structure, diversity and heterozygosity across multiple spatial scales in *A. lyrata*

Previous studies have shown strong population structure across the range of *A. lyrata* with the origin of diversity in Central Europe [5, 48, 49], a bottleneck in North American populations [46], and additional bottlenecks and independent origins of North American selfing populations [54, 56]. Using only 2–4 individuals per population, but multiple populations per region and analyses based on RAD loci, we found remarkably similar results to those based on whole genome re-sequencing of fewer populations per region but more individuals per population (e.g. [5]). For example, we observed the separation of a North European and Eurasian/Amphiberingean genepool from a genetically highly diverse Central European genepool [61, 62], which was estimated based on whole chloroplast genome sequence data to have occurred approximately 500,000 years ago [16]. This northern genepool must have undergone severe Pleistocenic differentiation and multiple range expansion and contractions, as also indicated by complex reticulate and cross-species evolutionary processes in the Russian Far East and the amphiberingean area [61–63]. Interestingly, we could also clearly differentiate most populations within the three Scandinavian countries (Fig. 2) and found evidence for isolation by distance within countries (Additional file 1: Figure S3), suggesting that genetic differentiation among populations within regions is relatively strong. Similarly, we found stronger genetic differentiation among the *A. l. lyrata* populations around the Great Lakes than observed using microsatellites (Fig. 3b, c), even though we only genotyped half the number of individuals per population with RAD-seq. The RAD markers thus clearly resolved population genetic structure within and among species to set a framework for interpreting patterns of selection.

### Detecting signatures of selection at multiple spatial scales

#### The effects of strategies for filtering and outlier detection

Overall patterns of polymorphism and heterozygosity were not substantially affected by filtering to allow inclusion of RAD loci that were not present in all individuals (Additional file 1: Table S2). This is consistent with recent work showing that varying the amount of missing data had limited effects on summary statistics estimated using empirical and simulated data [64]. In our study, relaxed filtering to consider loci present in at least 50% individuals had limited effect on the power to detect regions under balancing selection compared to requiring loci to be present in all individuals (Table 1b), but resulted in higher nucleotide diversity and expected heterozygosity (Additional file 1: Table S2), suggesting inclusion of more polymorphic gene regions.

Consistent with other studies, our results suggest that outlier analyses based on genomic smoothing are preferable to using single RAD loci when looking for genes showing elevated polymorphism. For example, using RAD-seq data and the *Stacks* smoothing algorithm, Hohlenlohe et al. (2010) similarly found evidence for high diversity and reduced F_ST_ at the stickleback Major Histocompatibility locus, a locus known to show signatures of balancing selection in a wide range of taxa. Using smoothing over multiple RAD loci can minimise the risk of identifying high diversity genomic regions based on an incorrectly assembled individual locus and thus increase the power to distinguish outliers. Additionally, given the importance of accurately calling SNPs for assessing variation in diversity, we used a 10× minimum coverage per locus for calling heterozygotes and used the *rxstacks* module from the Stacks pipeline (http://catchenlab.life.illinois.edu/stacks/comp-v1/rxstacks.php) to exclude and, where possible, correct likely sequencing errors from our RAD dataset. Finally, our study demonstrates the power of this smoothing approach even with relatively sparse marker density.

#### Disease resistance candidates are associated with genomic regions of elevated diversity

Nucleotide binding site-leucine rich repeat proteins (NLRs), are a major class of Resistance Gene Analogs (RGA) known to be good at generating allelic diversity, and such diversity is important for recognizing a diverse pool of potential pathogen effectors [39, 65]. NLRs are a well-annotated gene family in *A. thaliana* and around two-thirds of known *R-*genes in *A. thaliana* have a matching gene in *A. lyrata.* We found, as predicted, that the density of NLR loci in *A. lyrata* was significantly higher in genomic regions of elevated allelic diversity than other regions of the genome and that this result was consistent across different sample groups of *A. lyrata* (Table 2). By contrast, the density of a related set of RGAs*,* LRR-SLKs, was not higher in regions of elevated diversity, as might be expected from their involvement in more conserved ligand interactions [35]. This is consistent with whole genome sequencing studies, with the NLR class of disease resistance genes in *A. thaliana* found to be at higher frequency in regions of elevated diversity and SLKs proportionately less well-represented in high diversity regions [38]. Similarly, using shotgun sequence fragments, Cork & Purugganan (2005) [66] identified genomic regions of high synonymous diversity across *A. thaliana* ecotypes, and found one containing a predicted TIR-NBS-LRR gene.

An advantage of our broad sampling of *A. lyrata* subspecies was that we could identify candidate loci present in high diversity genomic regions in multiple independent sample groups. In total, 37.9% of the 137 NLRs were present in high diversity regions in at least two sample groups, and 8.8% across all three sample groups (Fig. 5). Furthermore, NLR genes shared between North American and European *A. lyrata* included disease resistance candidates known to show elevated levels of polymorphism in *A. thaliana* (Additional file 1: Table S6)*;* for example, *RPP7* and *RPP13* involved in strain specific resistance to the downy mildew pathogen *Hyaloperonospora arabidopsidis* [40, 67], and a homolog of *RLM1B* involved in fungal resistance [40, 68]. Interestingly, one gene was annotated as a paralog of *WRR4* (At1g56540) and in the same cluster as *WRR4* (At1g56510) [60]*. WRR4* confers broad spectrum resistance to the oomycete rust pathogen *Albugo candida* was in a genomic region of elevated diversity in all outcrossing groups in our study, and also shows elevated diversity in both *A. thaliana* [40] and *Capsella* sp. [57]. This locus showed higher nucleotide diversity based on Sanger sequencing for a more extensive sampling of *A. l. lyrata* from the Great Lakes region when compared to average nucleotide diversity across a conservative set of RAD loci present in all individuals [51]. Several other *R-*genes thought to be influenced by balancing selection were only present in genomic regions of significantly elevated diversity in one of the population groups; for example, two genes recognising *Pseudomonas* bacteria in the NA-O samples, *RPM1* [69, 70] and *RPS2* [59, 71]. This might not be unexpected since these are members of large gene families where there might be variation in the number of copies retained between populations [42]. Nevertheless, together with our results, these studies support the hypothesis that the evolution of NLR genes may be driving patterns of elevated polymorphism in some parts of the genome.

One criticism of the use of RAD markers is that the resolution of such loci to detect individual genes under selection is low, particularly when a gene has been under long-term selection and recombination has reduced linkage to surrounding sequences [18]. By reducing the number of individuals a locus had to be present in, or reducing the read coverage per locus, we could have increased marker density, but at the cost of reduced accuracy in estimating certain statistics across the genome. For example, low sample sizes are a particular problem for estimating F_ST._ In any case, the relatively high density of individual markers, particularly in the more variable outcrossing sample groups (on average every 11.6Kbp or 13.5Kbp), means that our resolution with respect to individual genes was relatively high. For example, even for the NA-S samples, individual high diversity individual RAD loci could be identified in the vicinity (+/− 2500 bp) of annotated *R-*genes (see NA-S single outlier overlap with smoothed outlier windows; Additional file 1: Table S4).

Our study also shows the potential for using genome scan data in a reverse genetics approach to identify loci in *A. lyrata* that may be generating allelic diversity for disease resistance. Targeted sequencing of candidate genes would of course be necessary to confirm the elevated patterns of diversity, and gene knock-outs of the corresponding *A. lyrata* loci could be used to see whether this reduces fitness under field conditions, in particluar when associated with elevated densities of pathogenic microbes. Most of the *R-*genes we focus on in this paper are examples from *A. thaliana* that have had reports of a functional allele conferring resistance to a bacterial, fungal or oomycete pathogen (Additional file 1: Table S6, S7). However, we also identify NLR loci for which there is no evidence in other studies for an interaction with a pathogen (Additional file 1: Table S6/S7), making these interesting novel candidates for further study.

#### Multiple population genetic statistics can help detect genome-wide signatures of balancing selection

In addition to elevated polymorphism, balancing selection may also leave signatures of reduced genetic differentiation among populations and shifts in the allele frequency spectrum towards intermediate frequencies [22, 23]. A key aim of our broad sampling approach was to ensure high neutral F_ST_ among sample groups and therefore increase our power to detect this signature of balancing selection (e.g. [24]). However, we found relatively few genomic regions (and associated NLR candidates) showing both elevated diversity and reduced F_ST_ among diverged sample groups, with relatively more genomic regions showing both elevated polymorphism and elevated F_ST_ (Fig. 6; Additional file 1: Figure S10; Additional file 1: Table S7). This is similar to recent results from a study on Anopheles mosquitos using a comparable strategy to our own (Kamdem et al. (2017) [4]. In their case, the limited evidence for balancing selection could result from low levels of background F_ST_ within the two mosquito species compared, but our sampling of a small number of individuals from strongly differentiated populations suggests that the rarity of these signatures of balancing selection could be more than just lack of power to detect outliers. A genome-wide screen of a population of *Drosophila melanogaster* also only found 30 of 13,900 protein-coding genes to show signatures of balancing selection based on elevated Tajima’s D and Watterson’s estimator Ø_W_ [17]. Thus, balancing selection might be a relatively rare form of selection shaping genome-wide patterns of polymorphism.

The higher concordance between elevated diversity and elevated F_ST_ could represent the random fixation of diverged NLR alleles in the different subspecies by genetic drift, or selection maintaining diverged alleles in different geographic locations. Selection by distinct pathogen communities in different geographic locations could result in increased differentiation between geographic regions or populations, and also elevate diversity across sample groups at these loci [72]. Such patterns of diversifying selection have been described for MHC class II genes in amphibians (e.g. [73]), as well as at effector proteins that interact with target host plant defense proteins in the stem rust fungus, *Puccinia graminis* [74]. However, despite high allelic divergence and diversity at NLR genes in plants, there is limited evidence for this translating into increased divergence among natural populations [40]. Here, we identify a number of interesting candidate loci in regions of the genome showing signatures of diversifying selection. For example a downy mildew resistance locus *RPP8,* showed elevated polymorphism, F_ST_ and Tajima’s D across EU-N countries (Additional file 1: Table S7). In *A. thaliana, RPP8* also shows elevated levels of polymorphism [71, 75] and a long evolutionary history consistent with long-term balancing selection [39, 76]. However, many candidate NLR genes identified in our study have not yet been functionally described in *A. thaliana* (Additional file 1: Table S7) and would require further functional testing.

In contrast to lack of evidence for reduced population structure in regions of elevated diversity, there was a large degree of overlap between genomic regions of significantly elevated polymorphism and elevated Tajima’s D (shifts to intermediate allele frequencies). For example, a *RPP8* homolog and a paralog of *WRR4* were present in such regions, along with genes annotated as part of the *RPP7* and *RPP1* gene clusters (Additional file 1: Table S7)*.* Specifically, the *WRR4* paralog showed elevated diversity in both EU-N and NA-O sample groups, as well as elevated D across the NA-O group and in Norweigian samples. Interestingly, both this *WRR4* paralog and *At1g59780* (part of the *RPP7* gene cluster [67]), show elevated diversity and high Tajima’s D in *A. thaliana* [40]. Furthermore, the *WRR4* paralog exhibits signatures of balancing selection, high diversity and reduced F_ST_, in both *Capsella grandiflora* and *C. rubella* [57]. Thus, although the number of loci resolved was low, detection of genes under balancing selection may be stronger based on shifts in allele frequency spectra than reduced differentiation. However, it should be noted that such an overlap between elevated polymorphism and Tajima’s D could partly result from the non-independence of these statistics, but also from the presence of fine-scale genetic structure within our sample groups [25]. Our strategy to sample broadly across populations rather than intensively within populations while useful for detecting population structure and increased polymorphism might not have been as appropriate for detecting shifts in allele frequency spectra due to selection rather than demography.

## Conclusions

By sampling a small number of individuals within populations across multiple spatial scales, we confirmed and extended previous predictions about both broad- and fine-scale phylogeographic differentiation in *A. lyrata*. Our smoothed outlier analyses based on relaxed filtering of missing data demonstrated that high throughput genotyping approaches, such as RAD-seq, retain power to detect genes in regions of the genome showing signatures of different types of selection. We identified genomic regions of elevated polymorphism containing disease resistance candidates known to be influenced by balancing selection in *A. thaliana*. Furthermore, within regions showing classical signatures of balancing selection (high diversity, low differentiation or intermediate allele frequencies), we identified novel candidate disease resistance genes, which have not been previously described as balanced polymorphisms in other taxa. Interestingly, we identified relatively few signatures of classical balancing selection, specifically genomic regions showing both high diversity and reduced differentiation. While this could result from a lack of power to detect reduced F_ST_ with RAD markers, the high average differentiation between and within *A. lyrata* subspecies instead suggests that this mode of selection could be relatively rare, even for a priori candidates such as disease resistance loci. In fact, candidate disease resistance loci in *A. lyrata* were mostly located in regions showing both elevated polymorphism and elevated differentiation, suggesting that diversifying selection may play a more important role than expected in pathogen resistance evolution across broad geographic ranges. Overall, our study provides insight into the relative frequency of signatures of balancing and diversifying selection across the genome, but also identifies candidate genes that could be targeted with whole gene sequencing to further investigate these alternative modes of selection.

## Methods

### Sampling of *Arabidopsis lyrata* subspecies

***Arabidopsis lyrata ssp. lyrata*** Seeds collected from individual plants were sampled at 12 sites (four selfing, and eight outcrossing populations) across the North American Great Lakes region in the summer of 2011 (Fig. 1a; described in detail in Buckley et al. 2016 [51]). A population previously described as mixed mating (TSSA) was grouped with the outcrossing samples because it shows similar patterns of diversity and heterozygosity to other outcrossing populations [51, 54]. In addition, seeds sampled in 2004 were included for one selfing population (TC), because no seeds were found in the 2011 field season. Seeds from six individual maternal plants from each population were germinated in a growth cabinet (16:8 h day:night cycle; 20 °C:16 °C; 80% humidity), and DNA extracted from leaves from one individual from each of four separate maternal families per population. For TC, individuals germinated from only one maternal family, so in total there were 49 *A. l. lyrata* individuals from different maternal families (Additional file 1: Table S1a).

***Arabidopsis lyrata ssp. petraea*** Samples from European populations were collected from individual plants in the summer of 2007, apart from the leaf tissue from German and Austrian plants, which was collected in 2012 (Additional file 1: Table S1b). Although *A. l. petraea* is known to vary in ploidy across its range [49], only confirmed diploid populations were included [63]; Mable, unpublished). Seeds from the Scottish population were generously provided by Elizabeth Bourne. Seeds were germinated and grown in the same growth cabinet conditions as described for *A. l. lyrata,* and DNA extracted from leaves of one individual from 2 to 3 maternal families per population (Fig. 1b; Additional file 1: Table S1b). From central Europe, DNA was extracted from field-collected leaf tissue from 3 individuals from Germany and 2 from Austria. In total, 42 diploid individuals from *A. l. petraea* were used (Fig. 1b).

### DNA extraction, RAD library preparation and sequencing

Fresh leaf tissue was dessicated using Drierite drying agent (WA Hammon Drierite Co. LTD, Xenia, US). DNA was extracted following the protocol of the Qiagen DNeasy plant mini kit (Qiagen, Manchester, UK), from dried leaf tissue, disrupted using a Fastprep machine and Fastprep lysing matrix A tubes (MP Biomedicals, Santa Ana, California, USA). DNA extractions from multiple leaves of an individual were pooled to obtain a minimum of 2 μg per individual (at least 10 ng/ul). DNA quantification and quality checks were performed using a Nanodrop ND1000 spectrophotometer and 2% agarose gel electrophoresis. Final quantifications were performed using high-sensitivity double-stranded DNA assay kits for the Qubit 2.0 Fluorometer (Life technologies Ltd., Paisley, UK).

DNA from the 91 individuals was sent to Edinburgh Genomics (Edinburgh, UK) for 100 bp paired-end Restriction Associated DNA sequencing. DNA was digested using *Pst*1*,* which is not sensitive to methylation, and was predicted to produce 64,056 RAD loci across the 207 Mbp *A. lyrata* genome. Each individual was tagged using barcodes differing by at least 2 bp. Three *Pst*1 libraries for the 49 North American individually barcoded samples were optimized and sequenced using one lane of an Illumina HiSeq 2000 sequencer. The process was then repeated for the 42 European samples.

Raw 100 bp reads were demultiplexed using the individual barcodes. FastQC (Babraham informatics, Cambridge, UK) was used to check read quality and to count total reads per individual. Reads were filtered to remove those with uncalled bases and those of low quality using the flags –c and –q in the *Stacks* program *process_radtags* [58]*.* A window that is 15% of the size of the read length is moved across each read and removes the read if the average quality score within the window drops below a minimum phred score of 10. The reads were trimmed to 92 bp to remove the ID tags. Using fastQC to inspect read quality after the above-described read filtering revealed that average read quality score showed a clear peak at 38 in all samples, and that the median base quality rarely falls below a quality score of 32 (with the lower interquartile values rarely dropping below 30). Therefore, the reads were of high quality for use in further analyses.

### Alignment of Illumina reads to reference genome and RAD locus assembly

Reads were aligned to the *A. lyrata* ssp. *lyrata* reference genome [45] (Phytozome version 1.0) using bowtie version 0.12.9 [77]. Specifically, we aligned reads with only one genome location (−m 1 in bowtie) to the eight chromosomes of *A. lyrata,* ~ 197Mbp in length, using default parameter conditions (seed length of 28, maximum of two mismatches allowed within seed). The reference-aligned reads were then processed by *Stacks v1.32* [58, 78] in order to assemble RAD loci for each individual by genomic position, and then call genotypes using a maximum likelihood framework [7, 78]. We also used the error correcting module *rxstacks* to reduce the potential for sequencing errors to inflate diversity estimates. Specifically, this module removed a small number of assembled RAD loci (on average 167 per sample) with highly negative log-likelihoods that likely represent sequencing errors and removes erroneous SNP calls (affecting only 0.01% loci on average; see Additional file 1: methods for details). To strike a balance between accurately calling heterozygotes and retaining large numbers of reads (and loci), we adopted a minimum threshold of 10 reads per individual per locus (Additional file 1: Figure S12). The proportion of heterozygous nucleotide sites was unaffected by increasing this threshold beyond 10 (Additional file 1: Figure S12b).

Of the 64,056 predicted loci, the majority (77.1%) were recovered in the catalog of all loci in all individuals. However, many of these loci are not present in all individuals. Approximately 51.7% (European samples) or 60.6% (North American samples) loci were assembled per individual (out of the 64,056 predicted), reducing to 40.7 and 42.5% after removing those loci with fewer than 10 reads per locus. The majority of loci were removed at the *populations* stage, when filtering was applied to select only loci present in all or at least 50% individuals. Only 13.9% of all predicted loci were present in all samples, increasing to 33.8% of all predicted loci when up to 50% missing data were allowed within the sample sets.

### Genetic structure, diversity and heterozygosity across multiple spatial scales in *A. lyrata*

Nucleotide diversity was estimated as the average number of nucleotide differences for all pairwise comparisons of sequences within a regional group, averaged across RAD loci and divided by the length of the RAD sequence (92 bp), equivalent to the nucleotide diversity estimate of Nei (1987) [79]. Expected heterozygosity (with alleles equivalent to 92 bp RAD haplotypes) was also estimated [79]. Observed heterozygosity was estimated as the number of loci heterozygous for the 92 bp RAD haplotype divided by the total number of loci observed in that individual.

We then used those loci present in all 91 individuals to determine the degree of genetic structuring within and between regions. RaxML v8 [80] was used to reconstruct a Maximum likelihood tree based on genome-wide SNPs and IUPAC codes were used to indicate heterozygous sites for each individual; invariant sites were not considered. A General Time Reversible (GTR) model with gamma distributed rate heterogeneity was used to model nucleotide substitutions. A bootstrap analysis (1000 pseudoreplications) was conducted to assess confidence in branching relationships.

A PCA analysis was also conducted to visualise genetic clustering across all the samples using polymorphic loci present in all individuals. We used RAD haplotypes (92 bp alleles) rather than SNPs for these analyses. RAD haplotypes identified by *Stacks* were converted to alleles in Genepop format using the *haplotypes.tsv Stacks* file and a custom R script by Mark Ravinet (https://github.com/markravinet/haplotype_to_genepop). Using the R packages “*adegenet*” [81, 82] and “*ade4*” [83] locus allele frequencies were centred around zero to ensure variances were comparable across loci. The first two principal components were plotted against each other for visualising clustering of samples. Separate PCAs were conducted to assess clustering among Northern European populations (*N* = 37, using polymorphic loci present in all of those individuals), among populations within the three different Scandinavian countries (Norway, Sweden and Iceland), and across all North American Great Lakes samples (*N* = 49). For North American samples we also compared patterns of genetic structure inferred using RAD loci to genetic structure inferred using eight microsatellites (data from Foxe et al. 2010 [54]).

Finally, we used RAD loci present in all individuals to estimate IBD relationships for regions with sufficient sampling of populations, specifically all eight North American outcrossing populations and the five populations in each of Norway, Sweden and Iceland. Pairwise F_ST_ (Weir & Cockerham) values were estimated using the R program “*diveRsity*” [84], and geographic distances among population pairs was calculated using the *earth.dist* function in the “fossil” package [85]. Finally, multiple regression on matrixes (*MRM* function in package “ecodist”; [86] was used to test the significance of the IBD relationships. Isolation by distance relationships and plots were based on the standardised genetic distance estimate Fst/1-Fst.

### Detecting signatures of balancing selection at multiple spatial scales

#### Defining population subsets at multiple spatial scales

We used several complementary approaches to examine genome-wide patterns of polymorphism and divergence and help detect signatures of balancing selection, focusing analyses on three different subsets of samples from the two subspecies. The following sets of samples were defined:

**NA-S:** We tested whether excesses of heterozygosity and diversity in genes under balancing selection can be detected against an overall low background level of polymorphism using individuals from five selfing populations of *A. l. lyrata* (*N* = 17; with only one individual for TC; Fig. 1a).

**EU-N and NA-O**: We then identified regions of elevated polymorphism that overlap with regions of reduced population structure or intermediate allele frequencies (elevated Tajima’s D) using two sets of outcrossing populations sharing a recent demographic history and similar geographic coverage (area and distance among samples). For *A. l. petraea* we used samples from three Northern European countries (**EU-N**; 5 populations from each of Iceland, Norway and Sweden; 2–3 individual per population, *N* = 35; Fig. 1b) and for *A. l. lyrata* we used all North American Great Lakes outcrossing samples (**NA-O**; 8 populations, four individuals per population, *N* = 32; Fig. 1a).

**ALL-O**: We then compared divergent lineages to increase our power to test for signatures of reduced population structure that overlap with genomic regions of elevated polymorphism. We compared all *A. l. lyrata* NA-O samples and all *A. l. petraea* samples, including the EU-N samples, but also two samples from Scotland and five samples from Central Europe (Fig. 1b), the latter thought to be the ancestral origin of diversity of the species [48].

#### Filtering threshold decisions and population genetic diversity statistics

For the population genomic analyses, we focused on loci with up to 50% missing data (present in a minimum of 50% of individuals in a sample group: NA-S, NA-O, EU-N or ALL-O). We also tested the consequence of this relaxed filtering (< 50% missing data vs 0% missing data) for the detection of candidate loci using the NA-S samples.

For estimating observed heterozygosity, nucleotide diversity and expected heterozygosity, we used variation in RAD haplotypes (linked set of variants within each 92 bp RAD locus) rather than considering individual SNPs at RAD loci. This strategy was used because highly polymorphic genes might be expected to have multiple SNPs within a 100 bp region, so allelic diversity may be particularly high and would be missed just by using a SNP-based approach. Both π and *H*_*e*_ are calculated by the program *Stacks*, whereas *H*_*o*_ was estimated from *Stacks* output. Specifically, observed heterozygosity (*H*_*o*_) was estimated as the proportion of heterozygous individuals at a RAD locus out of the total number of individuals genotyped at that locus. Nucleotide diversity (π) was estimated as the average number of nucleotide differences for all pairwise comparisons of sequences within the set of samples, divided by the length of the RAD sequence (92 bp), which is equivalent to the estimate of Nei (1987). Expected heterozygosity (gene diversity, *H*_*e*_) was also estimated (Nei, 1987, p.180) for more direct comparison with *H*_*o*_.

To detect genomic regions showing high diversity, we adopted a smoothing algorithm implemented in *Stacks* [7] to generate smoothed distributions of 92 bp haplotype-level π and *H*_*e*_, as described in Hohenlohe et al. (2010). Each smoothed window is defined by the value σ, where a window 6σ in length is centred on a polymorphic RAD locus. The width of the window was set for different sampling groups to encompass on average 30 RAD loci. The average diversity measure of RAD loci within this window is then weighted by distance to the RAD locus (3σ in each direction), so RAD loci in the centre of the window more strongly influence overall window polymorphism. Since observed heterozygosity would not be as meaningful across genes (i.e. because it is a binary rather than a quantitative trait), only π and *H*_*e*_ were compared using the smoothing approach. Bootstrap resampling (with 1,000,000 pseudoreplicates) was used to test for windows showing significantly higher diversity than the genome-wide average (as described in [7]). Windows of significantly elevated diversity were identified using the relaxed threshold of *p* < 0.05, as we were primarily interested in comparing overlapping windows among sample groups rather than robustly identifying all significantly elevated diversity regions per se. When adjacent windows of significantly elevated diversity overlapped (i.e. separated by less than the value of σ), they were considered as a single genomic region of significantly elevated diversity for annotation. The number and ID of genes in regions of elevated diversity were then compared among the different sample groups (NA-S, EU-N, NA-O) to look for shared candidate loci.

#### Identifying candidate genes in smoothed candidate regions

To test the relative ability of each of the above strategies to detect regions under balancing selection, we focused on a test panel of *A. lyrata* genes annotated as associated with disease resistance. This set included all annotated Nucleotide Binding Site-Leucine Rich Repeat (NLR) genes, plus some additional loci (Additional file 1: Table S5). We subsequently refer to these as *A. lyrata* NLR genes. All *A. lyrata* NLR genes within a genomic region of interest (showing either elevated diversity alone, or elevated diversity combined with reduced F_ST_ or elevated Tajima’s D) were extracted from a downloaded gff file (*A. lyrata*_107_v1.0_gene_details.gff from Phytozome v107).

We also looked for evidence of balancing selection (elevated diversity, intermediate allele frequencies, reduced genetic differentiation among populations and allele sharing among diverged lineages) within this set of NLR genes in Brassicaceae species. In total 17 of these genes represented clear a priori candidates for being under balancing selection [39–41, 57, 66, 69, 71, 75, 87–90], so these represent strong a priori candidates to be detected by our approach (summarised in Additional file 1: Table S6).

We used a randomisation test to test the prediction that the density of NLR loci is higher in windows of significantly elevated diversity than outside these windows. The randomisation test estimated whether the average number of NLR genes per smoothed diversity window (+/− 1 x σ either side of each polymorphic locus) was significantly greater in windows of significantly elevated diversity compared to windows of non-significantly elevated diversity. Windows were labelled as “high diversity” or “not high diversity”, then this label was randomly permuted (without replacement) and the difference in density of NLR genes between the two categories recalculated. This was repeated 1000 times to produce a null distribution of differences to which the observed difference is compared. The *p*-value indicated the proportion of simulated differences that were greater than the observed difference. For comparison to the NLR genes, we used a set of Leucine Rich Receptor- Receptor-like kinases (LRR-RLKs), which are not expected to be at higher frequency in regions of elevated diversity [35]. We used a list of 223 LRR-RLK genes present in *A. thaliana* [91] and then searched for *A. lyrata* genes annotated as these loci, resulting in 253 genes present in the *A. lyrata* genome assembly.

#### Identifying loci showing elevated observed heterozygosity and diversity in a low variation background (NA-S samples)

Given the low overall levels of heterozygosity and diversity across the selfing (NA-S) samples, we looked for candidate genes in regions of significantly elevated diversity (both nucleotide diversity and expected heterozygosity), initially based on single outliers and then based on smoothing across multiple RAD loci. The single outlier approach involved identifying loci with the highest 1% values of π, *H*_*e*_ and *H*_*o*_ (described in the Additional file 1). Smoothed windows that showed either a smoothed estimate of expected heterozygosity or nucleotide diversity with significance p < 0.05 were considered as ‘high diversity windows’. Each window was 900Kbp wide (σ value of 150Kbp), as the low number of polymorphic loci across the selfing samples meant a large window size was required to encompass on average 20 RAD loci (or on average 33 RAD loci when up to 50% missing data allowed). Neighbouring windows of significantly elevated diversity were then merged to form high diversity genomic regions and annotated NLR genes identified. For this set of samples, we also tested whether changing the amount of missing data (0% or 50%) affected the number of candidate regions and the number and IDs of NLR loci detected.

#### Identifying loci showing elevated diversity, reduced genetic differentiation and intermediate allele frequencies in outcrossing populations (EU-N, NA-O and ALL-O samples)

For North American outcrossing and Northern European samples we focused on elevated π and *H*_*e*_ to identify high diversity genomic regions*,* but also used evidence for balancing selection based on reduced population differentiation (F_ST_) and an intermediate site frequency spectrum (Tajima’s D). These analyses were based on RAD loci present in a minimum of 50% of individuals in a sample group (< 50% missing data). For the weighted smoothing analysis, window size was adjusted to contain about 30 RAD loci, using a σ value of 60Kbp for both EU-N and NA-O sample groups (total window size = 360Kbp). Regions of significantly elevated diversity were identified in both groups separately and annotated NLR genes identified.

We then identified genomic regions of elevated diversity that also overlapped with windows of reduced population differentiation (F_ST_) or an intermediate site frequency spectrum (positive Tajima’s D values) within each of the NA-O and EU-N sample sets. F_ST_ was calculated in *Stacks* using RAD haplotypes and AMOVA-based F_ST_ estimates [92], based on equations for allelic data described in [93]. F_ST_ was estimated within smoothed windows of identical size to the smoothed diversity estimates. We calculated pairwise F_ST_ among sample groups clearly separated in the RaXML tree, specifically among the three EU-N countries and among three clusters of the NA-O populations (three clusters of outcrossing populations: IND, SAK, SBD vs MAN, PIN, PCR vs TSS, TSSA; Fig. 1c). Windows with low F_ST_ values (in the bottom 5% of the distribution of all windows) whose genomic position overlapped with regions of significantly elevated diversity were identified.

Tajima’s D could not be calculated using *Stacks* and was therefore calculated using individual SNPs (from a VCF output file from *Stacks*) in a sliding window analysis using the R package “*PopGenome*” [94]. Windows were 120Kbp (+/−σ) wide and shifted by 12Kbp each time (the average approximate distance between RAD loci) and Tajima’s D estimated using polymorphic sites in that window. From the distribution of Taijam’s D values across all windows, we selected 5% of the windows with the highest Tajima’s D values (right hand tail of each histogram; Additional file 1: Figure S9). We compared the position of windows with these highest 5% of Tajima’s D values with the position of regions of significantly elevated diversity and identified all *A. lyrata* NLR genes in these overlapping regions. As D can be inflated by hidden genetic structure, these analyses were performed separately for each EU-N country in turn (Norway, Sweden and Iceland) and all NA-O samples together. NA-O samples were pooled as population divergence within the Great Lakes region was comparable to that observed within each Scandinavian country (Fig. 1c). In contrast to *Stacks*, the windows are based on genomic position (not centred on a RAD locus) and there was no option to weight loci based on distance to the centre of the window.

Finally, we compared populations of the strongly diverged *A. lyrata* subspecies. Windows of significantly elevated diversity across all outcrossing (All-O) samples and F_ST_ between subspecies were identified as described above, but using a reduced window size of 50Kbp due to the higher RAD marker density across this sample set. Overlap between elevated diversity and reduced F_ST_ windows were then examined for the presence of *A. lyrata* NLR genes.

## Additional file

Additional file 1: Contains supplementary methods and results, in addition to supplementary **Tables S1-S7** and supplementary **Figures S1-S12**. (DOCX 26587 kb)
