# Supplementary material for: Restriction associated DNA-genotyping at multiple spatial scales in Arabidopsis lyrata reveals signatures of pathogen-mediated selection
Source: BMC Genomics. 2018 Jun 27;19:496. doi: 10.1186/s12864-018-4806-7 (PMC6020377; doi:10.1186/s12864-018-4806-7)
Supplement: Supplementary file 1 — Contains supplementary methods and results, in addition to supplementary Tables S1-S7 and supplementary Figures S1-S12. (DOCX 26587 kb) [file 12864_2018_4806_MOESM1_ESM.docx]

**SUPPLEMENTARY INFORMATION**

**Restriction Associated DNA-genotyping at multiple spatial scales in *Arabidopsis lyrata* reveals signatures of pathogen-mediated selection**

**James Buckley, Eric B. Holub, Marcus A. Koch, Philippine Vergeer and Barbara K. Mable**

**SUPPLEMENTARY METHODS**

**Error correction by the rxstacks module**

Using the rxstacks module, we examined the frequency distribution of log-likelihoods for each locus in the assembled catalog to choose a log-likelihood threshold of a minimum of -20 to exclude loci with higher probability of sequencing errors (this step only removed on average 167 loci per sample). Nucleotide mismatches in a read were also corrected to the common variant if those sites were not called as statistically significant polymorphisms somewhere in the population. After error correction, *rxstacks* re-called sites as either homozygous or heterozygous using the ‘snp’ model type and a chi-square significance level of 0.1 for likelihood ratio tests. In our dataset, only a small percentage of uncalled sites were corrected to being homozygous or heterozygous (on average 0.0104%, ranging from 0.004% to 0.021%). This approach is conservative, so minimises the influence of assembly and sequencing errors on estimates of diversity.

**SUPPLEMENTARY RESULTS**

**Identifying outlier loci showing elevated observed heterozygosity and diversity in a low variation background**

Individual RAD loci with high values of observed heterozygosity (*H_o_*) *,* expected heterozygosity (*H_e_*) or nucleotide diversity (π) in the top 1% of loci were classified as high diversity outliers. We identified a combined total of 141 (0% missing) and 167 (< 50% missing) high diversity outliers (Table S4a). High diversity outliers often showed both high *H_e_* and π, but there was limited overlap of these loci with those displaying high *H_o_*. Only 3.6% (0% missing) and 5.4% (<50% missing) of the combined outliers showed both *H_o_*. and elevated π and/or *H_e_*. We excluded any highly polymorphic loci that showed evidence for multiple copies or residing in repetitive regions in *A. lyrata* (Table S4a). Specifically, we excluded any RAD loci with fixed heterozygosity across individuals and any showing >2 alleles within >1 individual. These loci may represent merged copy number variants or paralogs within *A. lyrata*.

Next, these high diversity RAD outliers were annotated using NCBI BLAST and the *A. thaliana* coding sequence database (TAIR10.29 downloaded from <http://plants.ensembl.org> on 02.11.15). All poor-quality BLAST matches with e-value <1.00E-10 were excluded. To annotate as many RAD loci as possible, we used a three-step strategy: a) megablast of 92bp RAD sequences against the TAIR coding sequence database to find unique matches; b) discontiguous megablast on the remaining unannotated RAD sequences; and c) megablast of a 5000bp of region around each RAD locus (2500bp either side extracted from the *A. lyrata* reference sequence) for loci still without any annotation or with multiple identical BLAST matches. Unique BLAST matches or best BLAST matches (with the lowest e-value when multiple BLAST matches were found) were allocated to RAD outliers where possible. We subsequently identified all BLAST matches where the TAIR ID was named as a “Disease resistance protein”. Where there were multiple indistinguishable TAIR matches (with identical e-values) for a RAD outlier, we examined all BLAST matches to find any named as a “disease resistance protein”.

Through this approach, we identified four RAD outliers annotated as disease resistance genes when allowing for 50% missing data, compared to just two using a locus set present in all individuals (Table S4a,b), which is consistent with our predictions that allowing missing data is important when examining loci showing high diversity and potentially complex patterns of segregation. Interestingly, none of the RAD loci showing both high diversity and observed heterozyogisty were BLAST annotated as disease resistance genes, although one did show significant similarity to a self-incompatibility (*S*-locus) region haplotype (on Chromosome 7), an interesting candidate region for being influenced by balancing selection. Nevertheless, despite identifying several interesting candidate genes, the single outlier approach was complicated by incorrectly assembled loci, particularly loci having fixed heterozygosity across all individuals or more than two alleles in multiple individuals at a locus. Furthermore, as the overall levels of polymorphism in the selfing data set were low, the density of polymorphic RAD loci was therefore too low to reliably pick out all interesting candidates using a single-outlier approach. We therefore adopted smoothing algorithms implemented in the program *Stacks* to identify genomic regions of interest from multiple RAD loci for all remaining sample groups.

**Additional Supplementary Information References**

Bittner-Eddy PD, Crute IR, Holub EB and Beynon JL. 2000. RPP13 is a simple locus in *Arabidopsis thaliana* for alleles that specify downy mildew resistance to different avirulence determinants in *Peronospora parasitica. The Plant Journal* **21:** 177-188

Botella MA, Parker JE, Frost LN, Bittner-Eddy PD, Beynon JL, Daniels MJ, Holub EB and Jones JDJ. 1998. Three genes of the *Arabidopsis* RPP1 complex resistance locus recognize distinct *Peronospora parasitica* avirulence determinants. *The Plant Cell* **10**: 1847-1860

Deslandes L, Olivier J, Theulieres F, Hirsch J, Fend DX, Bittner-Eddy P, Beynon J and Marco Y. 2004. Resistance to *Ralstonia solanacearum* in *Arabidopsis thaliana* is conferred by the recessive *RRS1-R* gene, a member of a novel family of resistance genes. *Proc Natl Acad Sci* 99: 2404-2409.

Gassmann W, Hinsch M, Staskawicz B (1999) The *Arabidopsis RPS4* bacterial-resistance gene is a member of the TIR-NBS-LRR family of disease-resistance genes. *The Plant Journal* **20**, 265-277.

Grant M, Godiard L, Straube E*, et al.* (1995) Structure of the Arabidopsis RPM1 gene enabling dual specificity disease resistance. *Science* **269**, 843-846.

Lee JM, Hartman GL, Domier LL, Bent AF. 1996. Identification and map location of TTR1, a single locus in Arabidopsis thaliana that confers tolerance to tobacco ringspot nepovirus. *Mol. Plant Microbe Interact*.; 9:729–735.

Lewis JD, Wu R, Guttman DS, Desveaux D. 2010. Allele-specific virulence attenuation of the *Pseudomonas syringae* HopZ1a type III effector via the Arabidopsis ZAR1 resistance protein. *PLoS Genet* 6:e1000894.

Lolle S, Greef C, Peterson K, Roux M, Jensen MK, Bressendorf S, Rodriguez E, Sømark K, Mundy J, Peterson M. 2017. Matching NLR Immune Receptors to autoimmunity in *camta3* mutants using antimorphic NLR alleles. *Cell Host & Microbe 21: 518-529.*

Noël L, Moores TL, van der Biezen EA*, et al.* (1999) Pronounced intraspecific haplotype divergence at the RPP5 complex disease resistance locu in Arabidopsis. *Plant Cell* **11**, 2099-2111.

Saucet SB, Ma Y, Sarris PF*, et al.* (2015) Two linked pairs of Arabidopsis TNL resistance genes independently confer recognition of bacterial effector AvrRps4. *Nat Commun* **6**, 6338.

Sinapidou E, Williams K, Nott L, Bahkt S, Tör M, Crute I, Bittner-Eddy P and Beynon J. 2004. Two TIR:NB:LRR genes are required to specify resistance to *Peronospora parasitica* isolate Cala2 in Arabidopsis. *Plant J* 38: 898-909.

Warren RF, Henk A, Mowery P, Holub E, Innes RW. 1998. A mutation within the leucine-rich repeat domain of the Arabidopsis disease resistance gene *RPS5* partially suppresses multiple bacterial and downy mildew resistance genes*. Plant Cell* 10:1439-1452.

**SUPPLEMENTARY TABLES**

**Table S1.** **Overview of *Arabidopsis lyrata* sampling locations from Europe and North America.** (a) the location of sites (and their respective code names) from which genotyped individuals were sampled for *Arabidopsis lyrata lyrata* from the Great Lakes region of eastern North America. The dominant mating system of *A. l. lyrata* populations is defined as O= outcrossing, or S = selfing. Note that TSSA has been previously described as mixed mating but was included with outcrossing populations in this study; (b) the location of sampling sites for *Arabidopsis lyrata petraea* from across Europe. All *A. l. petraea* populations are outcrossing. For both sets of samples, the year the individuals were sampled, the type of sample as either seeds or leaves collected from the field and number of samples genotyped from each population in the RAD study.

**a) *A. l. lyrata***

| **Site Code** | **Mating system** | **Location** | **Great Lake** | **Province/State** | **Latitude** | **Longitude** | **Year** | **Type** | **N** |
| --- | --- | --- | --- | --- | --- | --- | --- | --- | --- |
| KTT | S | Kitty Todd State Nature Preserve | Not relevant | Ohio, USA | N 41°37'14" | W 83°47'15" | 2007 | Seeds | 4 |
| LPT | S | Long Point Provincial Park | Erie | Ontario, Canada | N 42°34'47" | W 80°23'15" | 2011 | Seeds | 4 |
| MAN | O | Manitoulin Island | Huron/Georgian Bay | Ontario, Canada | N 47°39'54" | W 82°15'52" | 2011 | Seeds | 4 |
| PCR | O | Port Crescent State Park | Huron | Michigan, USA | N 44°00'15" | W 83°04'26" | 2011 | Seeds | 4 |
| PIN | O | Pinery Provincial Park | Huron | Ontario, Canada | N 43°16'08" | W 81°49'53" | 2011 | Seeds | 4 |
| PTP | S | Point Pelee National Park | Erie | Ontario, Canada | N 41°55'40" | W 82°30'51" | 2011 | Seeds | 4 |
| RON | S | Rondeau Provincial Park, | Erie | Ontario, Canada | N 42°15'41" | W 81°50'47" | 2011 | Seeds | 4 |
| SAK | O | Saugatauk Dunes State Park | Michigan | Michigan, USA | N 42°42'16" | W 86°12'31" | 2011 | Seeds | 4 |
| SBD | O | Sleeping Bear Dunes National Lakeshore | Michigan | Michigan, USA | N 44°56'20" | W 85°52'13" | 2011 | Seeds | 4 |
| TC | S | Tobermory cliffs, Bruce Peninsula National Park (BPNP) | Georgian Bay | Ontario, Canada | N 45°14'30" | W 81°31'03" | 2004 | Seeds | 1 |
| TSS | O | Tobermory Singing Sands, BPNP | Huron | Ontario, Canada | N 45°11'33" | W 81°35'02" | 2011 | Seeds | 4 |
| TSSA | O | Tobermory Singing Sands Alvar, BPNP | Huron | Ontario, Canada | N 45°11'27" | W 81°35'26" | 2011 | Seeds | 4 |

**b) *A. l. petraea***

| **Site code** | **Location (full name)** | **Country** | **Latitude** | **Longitude** | **Year** | **Type** | **N** |
| --- | --- | --- | --- | --- | --- | --- | --- |
| Berg | Berghamn | Sweden | N 62°49'32.7" | E 18°16'26.0" | 2007 | Seeds | 3 |
| Ham | Hamnslatten | Sweden | N 62°59'25.2" | E 18°32'04.7" | 2007 | Seeds | 2 |
| Not | Notsand | Sweden | N 62°36'30.9" | E 18°03'43.9" | 2007 | Seeds | 2 |
| Sjo | Sjoviken | Sweden | N 62°35'07.7" | E 18°01'03.1" | 2007 | Seeds | 2 |
| Ska | Skagsudde | Sweden | N 63°11'15.0" | E 19°00'49.9" | 2007 | Seeds | 3 |
| Ard | Ardal | Norway | N 61°20'0.33" | E 7°51'26.09" | 2007 | Seeds | 2 |
| Bov | Bovra | Norway | N 61°46'12.3" | E 08°25'08.8" | 2007 | Seeds | 2 |
| Lae | Laerdal | Norway | N 61°05'05.0" | E 07°31'11.4" | 2007 | Seeds | 3 |
| Sbo | Saebo | Norway | N 61°06'12.1" | E 07°25'18.5" | 2007 | Seeds | 2 |
| Spt | Spiterstulen | Norway | N 61°39'29.1" | E 08°25'37.7" | 2007 | Seeds | 2 |
| Faf | Fagrifoss | Iceland | N 64°18'01.58” | W 18°58'52.79" | 2007 | Seeds | 3 |
| NyN | Nyidalur.N | Iceland | N 64°43'13.97" | W 18°07'09.95" | 2007 | Seeds | 2 |
| NyS | Nyidalur S | Iceland | N 64°42'08.83" | W 18°10'03.68" | 2007 | Seeds | 2 |
| SaF | Sandfell | Iceland | N 64°04'21.0" | W 21°41'00.0" | 2007 | Seeds | 3 |
| SaT | Sandartunga | Iceland | N 64°07'24.1" | W 19°54'27"3 | 2007 | Seeds | 2 |
| CM | Coyles of Muick, Cairngorms | Scotland | N 56°57'49.73" | W 3°26'21.96" | 2007 | Seeds | 2 |
| VF | Veldensteiner Forst, Bavaria | Germany | N 49°41'11.17" | E 11°31'22.52" | 2012 | Leaves | 3 |
| BV | Bad Vöslau | Austria | N 47°57'53.31" | E 16°12'51.26" | 2012 | Leaves | 2 |

**Table S2**. **Polymorphism summary statistics for each major sampling group.** Average observed heterozygosity (*H_o_*), gene diversity (*H_e_*), and nucleotide diversity (π) in the four regional groups when loci had to be present in all (0% missing) or present in at least half of individuals (<50% missing). The number of individuals per sampling group (N) and total number of loci used to estimate heterozgosity and diversity statistics (N loci) are given.

|  |  |  |  |  |  |  |
| --- | --- | --- | --- | --- | --- | --- |
| **Group** | **N** | **Percentage missing** | **N loci** | ***H_o_*** | ***H_e_*** | **π** |
| NA-S | 17 | 0% | 5942 | 0.0103 | 0.1043 | 0.00131 |
|  |  | <50% | 16240 | 0.0136 | 0.1152 | 0.00148 |
| NA-O | 32 | 0 | 5942 | 0.0729 | 0.1189 | 0.00149 |
|  |  | <50% | 16240 | 0.0814 | 0.1344 | 0.00172 |
| EU-N | 37 | 0 | 5942 | 0.1489 | 0.2344 | 0.00318 |
|  |  | <50% | 16240 | 0.1477 | 0.2532 | 0.00356 |
| EU-C | 5 | 0 | 5942 | 0.2112 | 0.2758 | 0.00374 |
|  |  | <50% | 16240 | 0.2071 | 0.2837 | 0.00400 |

**Table S3.** **Variation in genome and locus coverage across the eight chromosomes and among sampling groups.** (a) The total number and density (per Mbp) of RAD loci with at least 10 x coverage on a chromosome on different chromosomes; (b) variation in sequencing coverage within and among different sample groups (North American selfing, NA-S; North American outcrossing, NA-O; Northern European, EU-N; Central European, EU-C) for sets of loci present in all (0% missing) or at least half of all sampled individuals (<50% missing). The number of individuals in a sample group (N) and mean, standard deviation, minium and maximum RAD-locus coverage in each sample set are given.

| **a)** |  | **0% missing** | | **<50% missing** | |
| --- | --- | --- | --- | --- | --- |
| **Chromosome** | **Length (Mbp)** | **N RAD loci** | **N RAD loci per Mbp** | **N RAD loci** | **N RAD loci per Mbp** |
| 1 | 33.60586 | 1108 | 32.97 | 3003 | 89.36 |
| 2 | 19.596877 | 528 | 26.94 | 1475 | 75.27 |
| 3 | 24.814041 | 845 | 34.05 | 2261 | 91.12 |
| 4 | 23.661599 | 632 | 26.71 | 1790 | 75.65 |
| 5 | 21.525117 | 601 | 27.92 | 1631 | 75.77 |
| 6 | 25.472354 | 876 | 34.39 | 2324 | 91.24 |
| 7 | 25.001329 | 767 | 30.68 | 2173 | 86.92 |
| 8 | 23.279169 | 585 | 25.13 | 1583 | 68.00 |
| **Total** | **196.956346** | **5942** | **30.17** | **16240** | **82.45** |

| **b)** | |  |  |  |  |  |  |
| --- | --- | --- | --- | --- | --- | --- | --- |
| **Sample group** | **N** | | **% Missing** | **Mean** | **SD** | **Min** | **Max** |
| NA-S | 17 | | 0 | 52.39 | 17.04 | 17.24 | 168.29 |
|  |  | | <50 | 43.04 | 20.52 | 11.33 | 247.27 |
| NA-O | 32 | | 0 | 64.18 | 21.13 | 20.03 | 185.38 |
|  |  | | <50 | 51.41 | 25.37 | 12.65 | 232.03 |
| EU-N | 37 | | 0 | 74.95 | 17.52 | 28.32 | 204.00 |
|  |  | | <50 | 62.00 | 23.09 | 12.91 | 286.89 |
| EU-C | 5 | | 0 | 60.03 | 14.11 | 17.60 | 176.80 |
|  |  | | <50 | 51.34 | 18.40 | 10.00 | 225.33 |

**Table S4**: **Summary of high diversity RAD outlier loci identified in selfing *A. lyrata* (NA-S) samples.** (a) the number of single RAD outliers (both total and polymorphic loci) detected for NA-S samples when loci present in all (0% missing) or present in at least half (<50% missing) were included in the analysis. The number of loci in top 1% of elevated nucleotide diversity (**π**), expected heterozygosity (***H_e_*)** and observed heterozygosity (***H_o_***) are given, as well as the number of RAD loci that were excluded due to evidence for incorrect locus assembly. Number of outlier loci that had a significant BLAST match to an *A. thaliana* gene were divided into those with unique BLAST hits or multiple BLAST hits (indistinguishable based on e-values), with the number of RAD loci annotated as NLR genes given for each BLAST set; (b) details about four RAD loci annotated as *Arabidopsis thaliana* disease resistance genes. The number of individuals in which this locus was observed, along with the frequency of different haplotypes at this locus (as characterised by the variant sites in the 92bp RAD sequence). Details of the RAD locus annotation with respect to *A. thaliana* and whether this outlier RAD locus is located in a genomic regino of significantly elevated diversity.

**a)**

|  |  |  | **Outlier Criteria** | | |  |  | **Unique BLAST match** | | **Multiple BLAST match** | |
| --- | --- | --- | --- | --- | --- | --- | --- | --- | --- | --- | --- |
| **% missing** | **N loci** | **N variant loci** | ***H_o_*** | ***H_e_*** | **π** | **Total N** | **Number excluded** | **N loci** | **NLR genes** | **N loci** | **NLR genes** |
| 1 | 6223 | 4327 | 49 | 58 | 62 | 143 | 2 | 121 | 2 | 11 | 0 |
| 0.5 | 10794 | 7256 | 79 | 65 | 71 | 184 | 17 | 131 | 4 | 7 | 0 |

| **b)** |  |  |  |  |  |  |  |
| --- | --- | --- | --- | --- | --- | --- | --- |
| **Chromosome** | **RAD locus position (bp)** | **Proportion missing data** | **N** | **Haplotype counts across samples** | **TAIR NLR gene annotation** | **Disease resistance protein class** | **Overlap with region of elevated diversity** |
| 2 | 3879340 | <50% | 12 | CA:7; CT:9; TA:8 | AT1G58602 | LRR and NB-ARC domains-containing | No |
| 6 | 18545618 | 0 or <50% | 17 | AAG:14; ATT:10; CAT:10 | AT4G08450 | TIR-NBS-LRR class family | Yes |
| 6 | 21698814 | 0 or <50% | 17 | CAA:10; CCG:13; TCA:11 | AT4G12010 | TIR-NBS-LRR class family | Yes |
| 7 | 24115168 | <50% | 13 | AACG:17; TGAC:9 | AT5G38350 | NBS-LRR class family | Yes |

**Table S5: *A. lyrata* loci annotated as disease resistance proteins in the *A. lyrata* reference genome.** These 206 loci, with ID number and best TAIR match from Phytozome version 1.1, form our NLR test panel. The location of the gene on the *A. lyrata* reference sequence is given, along with the RGA structural family, consisting mostly of Nucleotide Binding Site- Leucine Rich Repeat (NLR) genes, but also including several TIR-NBS and NB-ARC genes described as disease resistance loci in the *A. lyrata* reference genome.

| ***A. lyrata* gene ID** | **Best TAIR match** | ***A. lyrata* chromosome** | **Gene start (bp)** | **Gene end (bp)** | **TAIR description (annotated domains)** |
| --- | --- | --- | --- | --- | --- |
| 910841 | AT5G48620.1 | 1 | 4345321 | 4348320 | CC-NBS-LRR |
| 334514 | AT1G12220.2 | 1 | 4945426 | 4956021 | CC-NBS-LRR |
| 312255 | AT1G12280.1 | 1 | 4986226 | 4992556 | LRR and NB-ARC |
| 920066 | AT1G12220.2 | 1 | 4999423 | 4999969 | CC-NBS-LRR |
| 334861 | AT5G63020.1 | 1 | 6666431 | 6668282 | CC-NBS-LRR |
| 920497 | AT1G15890.1 | 1 | 6726892 | 6729351 | CC-NBS-LRR |
| 471969 | AT1G17600.1 | 1 | 7387052 | 7390362 | TIR-NBS-LRR |
| 471970 | AT5G40090.1 | 1 | 7390647 | 7391887 | TIR-NBS |
| 911573 | AT1G17615.1 | 1 | 7392985 | 7394218 | TIR-NBS |
| 912305 | AT1G27180.1 | 1 | 10834575 | 10841706 | TIR-NBS-LRR |
| 921508 | AT1G27180.1 | 1 | 10842854 | 10847412 | TIR-NBS-LRR |
| 473526 | AT1G33560.1 | 1 | 15541048 | 15544186 | CC-NBS-LRR |
| 913669 | AT2G14080.1 | 1 | 23034788 | 23046389 | TIR-NBS-LRR |
| 474150 | AT1G50180.1 | 1 | 26153627 | 26156565 | NB-ARC |
| 923884 | AT1G53350.1 | 1 | 28480753 | 28483801 | CC-NBS-LRR |
| 914883 | AT2G16870.1 | 1 | 31544323 | 31551475 | TIR-NBS-LRR |
| 914885 | AT1G58390.1 | 1 | 31570898 | 31577887 | CC-NBS-LRR |
| 914939 | AT1G56540.1 | 1 | 32339735 | 32344352 | TIR-NBS-LRR |
| 914947 | AT1G56540.1 | 1 | 32387793 | 32394095 | TIR-NBS-LRR |
| 914952 | AT1G56520.2 | 1 | 32425158 | 32425912 | TIR-NBS-LRR |
| 924349 | AT2G16870.1 | 1 | 32450108 | 32450552 | TIR-NBS-LRR |
| 474812 | AT1G56540.1 | 1 | 32458589 | 32467082 | TIR-NBS-LRR |
| 924354 | AT1G56540.1 | 1 | 32481643 | 32482090 | TIR-NBS-LRR |
| 915002 | AT3G44400.2 | 1 | 32907847 | 32922744 | TIR-NBS-LRR |
| 915006 | AT3G44400.1 | 1 | 32953852 | 32957010 | TIR-NBS-LRR |
| 907021 | AT1G63750.3 | 2 | 579910 | 583436 | TIR-NBS-LRR |
| 907022 | AT1G63730.1 | 2 | 585559 | 588751 | TIR-NBS-LRR |
| 337990 | AT1G63870.1 | 2 | 595318 | 596882 | TIR-NBS-LRR |
| 907024 | AT1G63870.1 | 2 | 600101 | 603822 | TIR-NBS-LRR |
| 474976 | AT1G63740.1 | 2 | 675019 | 678565 | TIR-NBS-LRR |
| 907036 | AT1G63730.1 | 2 | 678915 | 682264 | TIR-NBS-LRR |
| 338119 | AT1G62630.1 | 2 | 1600743 | 1604529 | CC-NBS-LRR |
| 907240 | AT1G63350.1 | 2 | 2019488 | 2025451 | CC-NBS-LRR |
| 907275 | AT2G16870.1 | 2 | 2263849 | 2267657 | TIR-NBS-LRR |
| 924912 | AT1G61310.1 | 2 | 2610768 | 2611092 | LRR and NB-ARC |
| 338250 | AT1G61190.1 | 2 | 2624930 | 2628149 | LRR and NB-ARC |
| 924914 | AT1G61310.1 | 2 | 2629832 | 2630520 | LRR and NB-ARC |
| ***A. lyrata* gene ID** | **Best TAIR match** | ***A. lyrata* chromosome** | **Gene start (bp)** | **Gene end (bp)** | **TAIR description (annotated domains)** |
| 315245 | AT1G61100.1 | 2 | 2711601 | 2718160 | TIR |
| 907475 | AT5G38850.1 | 2 | 3482698 | 3486134 | TIR-NBS-LRR |
| 907494 | AT1G58390.1 | 2 | 3614281 | 3617108 | CC-NBS-LRR |
| 907495 | AT1G59780.1 | 2 | 3617199 | 3617523 | NB-ARC |
| 907496 | AT1G59620.1 | 2 | 3617605 | 3619552 | CC-NBS-LRR |
| 907505 | AT1G59780.1 | 2 | 3679545 | 3702128 | NB-ARC |
| 475351 | AT1G59620.1 | 2 | 3879115 | 3888355 | CC-NBS-LRR |
| 907538 | AT1G59780.1 | 2 | 3901818 | 3902744 | NB-ARC |
| 907562 | AT1G58807.1 | 2 | 4040413 | 4045830 | CC-NBS-LRR |
| 907577 | AT1G58390.1 | 2 | 4153607 | 4156606 | CC-NBS-LRR |
| 907578 | AT1G59620.1 | 2 | 4160330 | 4161181 | CC-NBS-LRR |
| 338529 | AT1G63350.1 | 2 | 5036013 | 5036491 | CC-NBS-LRR |
| 907740 | AT5G22690.1 | 2 | 7616512 | 7619886 | TIR-NBS-LRR |
| 908051 | AT1G65850.1 | 2 | 10295848 | 10300693 | TIR-NBS-LRR |
| 908088 | AT1G66090.1 | 2 | 10671909 | 10674082 | TIR-NBS |
| 338955 | AT5G45220.1 | 2 | 12282796 | 12285075 | TIR-NBS-LRR |
| 908535 | AT1G69550.1 | 2 | 13828526 | 13831950 | TIR-NBS-LRR |
| 908894 | AT1G72840.1 | 2 | 15578883 | 15591830 | TIR-NBS-LRR |
| 908895 | AT1G72850.1 | 2 | 15592214 | 15593673 | TIR-NBS |
| 908896 | AT4G09360.1 | 2 | 15594164 | 15598541 | NB-ARC |
| 926653 | AT1G72870.1 | 2 | 15599239 | 15600847 | TIR-NBS |
| 908901 | AT1G72890.1 | 2 | 15611060 | 15612897 | TIR-NBS |
| 908906 | AT1G72950.1 | 2 | 15645182 | 15646394 | TIR-NBS |
| 909552 | AT5G17680.1 | 2 | 18542282 | 18550072 | TIR-NBS-LRR |
| 927380 | AT5G36930.2 | 2 | 18740094 | 18740602 | TIR-NBS-LRR |
| 477682 | AT3G04210.1 | 3 | 1406517 | 1408007 | TIR-NBS |
| 478023 | AT3G07040.1 | 3 | 2961540 | 2964400 | NB-ARC |
| 903839 | AT5G22690.1 | 3 | 3835858 | 3839130 | TIR-NBS-LRR |
| 478870 | AT3G14460.1 | 3 | 6076586 | 6080875 | LRR and NB-ARC |
| 478871 | AT3G14470.1 | 3 | 6082508 | 6085808 | NB-ARC |
| 479587 | AT3G20610.1 | 3 | 9060739 | 9061399 | Not specified |
| 930688 | AT1G27180.1 | 3 | 12909179 | 12909449 | TIR-NBS-LRR |
| 905786 | AT5G43470.2 | 3 | 13410042 | 13411451 | CC-NBS-LRR |
| 905841 | AT1G69550.1 | 3 | 13719374 | 13721395 | TIR-NBS-LRR |
| 906130 | AT1G69550.1 | 3 | 18616578 | 18619211 | TIR-NBS-LRR |
| 906175 | AT5G46450.1 | 3 | 19020384 | 19021804 | TIR-NBS-LRR |
| 906176 | AT5G46450.1 | 3 | 19027954 | 19031366 | TIR-NBS-LRR |
| 480565 | AT2G16870.1 | 3 | 21537390 | 21540965 | TIR-NBS-LRR |
| 932480 | AT4G36150.1 | 4 | 2787421 | 2791655 | TIR-NBS-LRR |
| 320248 | AT4G36150.1 | 4 | 2819772 | 2823793 | TIR-NBS-LRR |
| 900537 | AT5G41750.1 | 4 | 12208824 | 12212049 | TIR-NBS-LRR |
| 320641 | AT1G12220.1 | 4 | 12768477 | 12774611 | CC-NBS-LRR |
| 345479 | AT4G23510.1 | 4 | 17961393 | 17962685 | TIR-NBS-LRR |
| ***A. lyrata* gene ID** | **Best TAIR match** | ***A. lyrata* chromosome** | **Gene start (bp)** | **Gene end (bp)** | **TAIR description (annotated domains)** |
| 896621 | AT5G11250.1 | 5 | 3590971 | 3592916 | TIR-NBS-LRR |
| 896835 | AT5G46450.1 | 5 | 5223882 | 5228416 | TIR-NBS-LRR |
| 896915 | AT1G65850.1 | 5 | 5824233 | 5828028 | TIR-NBS-LRR |
| 897225 | AT5G11250.1 | 5 | 11213574 | 11217313 | TIR-NBS-LRR |
| 897229 | AT3G44400.1 | 5 | 11243892 | 11259147 | TIR-NBS-LRR |
| 897230 | AT3G44480.1 | 5 | 11266567 | 11280193 | TIR-NBS-LRR |
| 323301 | AT3G46730.1 | 5 | 12786866 | 12794517 | NB-ARC |
| 937390 | AT3G46530.1 | 5 | 12796169 | 12798517 | NB-ARC |
| 937702 | AT1G15890.1 | 5 | 14325183 | 14325449 | CC-NBS-LRR |
| 323710 | AT3G51560.1 | 5 | 15663348 | 15667448 | TIR-NBS-LRR |
| 485509 | AT3G51570.1 | 5 | 15667959 | 15672038 | TIR-NBS-LRR |
| 893040 | AT1G65850.1 | 6 | 4501442 | 4504847 | TIR-NBS-LRR |
| 488633 | AT5G17680.1 | 6 | 7290677 | 7294782 | TIR-NBS-LRR |
| 893705 | AT5G17880.1 | 6 | 7411281 | 7415196 | TIR-NBS-LRR |
| 941307 | AT5G17880.1 | 6 | 7422809 | 7423909 | TIR-NBS-LRR |
| 893712 | AT5G41550.1 | 6 | 7446614 | 7448322 | TIR-NBS-LRR |
| 893714 | AT5G17970.1 | 6 | 7450926 | 7453645 | TIR-NBS-LRR |
| 893756 | AT5G22690.1 | 6 | 7623722 | 7626916 | TIR-NBS-LRR |
| 893757 | AT5G18360.1 | 6 | 7627547 | 7630598 | TIR-NBS-LRR |
| 894148 | AT5G35450.1 | 6 | 9562491 | 9565486 | CC-NBS-LRR |
| 351902 | AT5G05400.1 | 6 | 17815874 | 17818531 | LRR and NB-ARC |
| 489751 | AT4G08450.1 | 6 | 18541263 | 18544722 | TIR-NBS-LRR |
| 895001 | AT2G14080.1 | 6 | 18882457 | 18884728 | TIR-NBS-LRR |
| 895002 | AT3G44630.3 | 6 | 18885580 | 18886038 | TIR-NBS-LRR |
| 895306 | AT4G11340.1 | 6 | 21108542 | 21111897 | TIR-NBS-LRR |
| 327329 | AT4G12010.1 | 6 | 21697931 | 21701993 | TIR-NBS-LRR |
| 888440 | AT4G36150.1 | 7 | 1972252 | 1976181 | TIR-NBS-LRR |
| 888441 | AT4G36140.1 | 7 | 1985558 | 1989059 | TIR-NBS-LRR |
| 354079 | AT4G27190.1 | 7 | 6336233 | 6338683 | NB-ARC |
| 492076 | AT4G27190.1 | 7 | 6340023 | 6342961 | NB-ARC |
| 945467 | AT4G26090.1 | 7 | 6811069 | 6813832 | NB-ARC |
| 889815 | AT4G23510.1 | 7 | 8049375 | 8061073 | TIR-NBS-LRR |
| 492529 | AT4G23440.1 | 7 | 8090276 | 8093333 | TIR-NBS |
| 889979 | AT5G11250.1 | 7 | 8898551 | 8901592 | TIR-NBS-LRR |
| 890180 | AT3G51570.1 | 7 | 9921012 | 9921293 | TIR-NBS-LRR |
| 946185 | AT4G36150.1 | 7 | 9921739 | 9925131 | TIR-NBS-LRR |
| 946227 | AT4G19520.1 | 7 | 10174371 | 10174626 | TIR-NBS-LRR |
| 890225 | AT4G19510.2 | 7 | 10175853 | 10179957 | TIR-NBS-LRR |
| 492950 | AT4G19050.1 | 7 | 10414241 | 10419988 | NB-ARC |
| 493178 | AT4G16990.1 | 7 | 11717600 | 11722784 | TIR-NBS |
| 890529 | AT4G16950.2 | 7 | 11732700 | 11738203 | TIR-NBS-LRR |
| 890654 | AT1G58400.1 | 7 | 12432537 | 12434570 | CC-NBS-LRR |
| 890967 | AT5G48770.1 | 7 | 14476211 | 14479427 | TIR-NBS-LRR |
| ***A. lyrata* gene ID** | **Best TAIR match** | ***A. lyrata* chromosome** | **Gene start (bp)** | **Gene end (bp)** | **TAIR description (annotated domains)** |
| 947018 | AT1G17610.1 | 7 | 14479513 | 14480360 | TIR-NBS |
| 493640 | AT1G51480.1 | 7 | 18868464 | 18871345 | CC-NBS-LRR |
| 891298 | AT3G25510.1 | 7 | 19883002 | 19888908 | TIR-NBS-LRR |
| 891371 | AT1G15890.1 | 7 | 20553780 | 20556335 | CC-NBS-LRR |
| 891414 | AT1G59780.1 | 7 | 20858994 | 20861897 | NB-ARC |
| 891488 | AT5G41750.1 | 7 | 21305401 | 21338529 | TIR-NBS-LRR |
| 891508 | AT5G41550.1 | 7 | 21441195 | 21444707 | TIR-NBS-LRR |
| 891510 | AT5G41550.1 | 7 | 21448572 | 21454901 | TIR-NBS-LRR |
| 891511 | AT5G41540.1 | 7 | 21455690 | 21457763 | TIR-NBS-LRR |
| 891572 | AT5G40910.1 | 7 | 21848238 | 21851832 | TIR-NBS-LRR |
| 891573 | AT5G40910.1 | 7 | 21852783 | 21856381 | TIR-NBS-LRR |
| 947767 | AT5G40100.1 | 7 | 22575056 | 22578524 | TIR-NBS-LRR |
| 493978 | AT5G40090.1 | 7 | 22583427 | 22584802 | TIR-NBS |
| 947840 | AT5G40090.1 | 7 | 23012144 | 23012798 | TIR-NBS |
| 891858 | AT5G38850.1 | 7 | 23595043 | 23596840 | TIR-NBS-LRR |
| 891927 | AT5G38350.1 | 7 | 24074465 | 24117335 | NBS-LRR |
| 891930 | AT5G38340.1 | 7 | 24125054 | 24128559 | TIR-NBS-LRR |
| 891933 | AT5G38350.1 | 7 | 24142367 | 24150920 | NBS-LRR |
| 891936 | AT5G38340.1 | 7 | 24156862 | 24160601 | TIR-NBS-LRR |
| 948180 | AT5G47250.1 | 8 | 303613 | 305110 | LRR and NB-ARC |
| 915416 | AT5G46450.1 | 8 | 871719 | 877677 | TIR-NBS-LRR |
| 915422 | AT5G46450.1 | 8 | 946821 | 956073 | TIR-NBS-LRR |
| 915423 | AT5G46450.1 | 8 | 958681 | 962749 | TIR-NBS-LRR |
| 915424 | AT5G46270.1 | 8 | 963755 | 968090 | TIR-NBS-LRR |
| 915425 | AT5G46450.1 | 8 | 970696 | 976059 | TIR-NBS-LRR |
| 915426 | AT5G46260.1 | 8 | 977734 | 981753 | TIR-NBS-LRR |
| 915427 | AT5G46270.1 | 8 | 989942 | 991645 | TIR-NBS-LRR |
| 494325 | AT5G46470.1 | 8 | 997049 | 1001951 | TIR-NBS-LRR |
| 494346 | AT5G46260.1 | 8 | 1085278 | 1090132 | TIR-NBS-LRR |
| 915586 | AT5G45260.1 | 8 | 2126189 | 2132181 | TIR-NBS-LRR |
| 948472 | AT5G45250.1 | 8 | 2132658 | 2135788 | TIR-NBS-LRR |
| 915589 | AT4G19510.1 | 8 | 2142399 | 2147078 | TIR-NBS-LRR |
| 915590 | AT5G45260.1 | 8 | 2149260 | 2157021 | TIR-NBS-LRR |
| 948480 | AT5G45240.1 | 8 | 2168790 | 2169117 | TIR-NBS-LRR |
| 915593 | AT5G45060.1 | 8 | 2201438 | 2205373 | TIR-NBS-LRR |
| 915594 | AT5G45050.1 | 8 | 2208065 | 2210267 | TIR-NBS-LRR |
| 915595 | AT5G45250.1 | 8 | 2210604 | 2212894 | TIR-NBS-LRR |
| 915597 | AT4G19520.1 | 8 | 2221712 | 2222877 | TIR-NBS-LRR |
| 948486 | AT5G45260.2 | 8 | 2223282 | 2227268 | TIR-NBS-LRR |
| 915598 | AT5G45250.1 | 8 | 2229850 | 2233642 | TIR-NBS-LRR |
| 915600 | AT5G45230.1 | 8 | 2249101 | 2255086 | TIR-NBS-LRR |
| 948493 | AT5G45240.1 | 8 | 2256516 | 2259035 | TIR-NBS-LRR |
| 948494 | AT5G45050.1 | 8 | 2263115 | 2265042 | TIR-NBS-LRR |
| ***A. lyrata* gene ID** | **Best TAIR match** | ***A. lyrata* chromosome** | **Gene start (bp)** | **Gene end (bp)** | **TAIR description (annotated domains)** |
| 948495 | AT5G45060.1 | 8 | 2281493 | 2286844 | TIR-NBS-LRR |
| 948499 | AT5G45240.1 | 8 | 2299544 | 2302249 | TIR-NBS-LRR |
| 494460 | AT5G45230.1 | 8 | 2305971 | 2311690 | TIR-NBS-LRR |
| 948501 | AT5G45240.1 | 8 | 2313092 | 2316297 | TIR-NBS-LRR |
| 948502 | AT5G45230.1 | 8 | 2330902 | 2332441 | TIR-NBS-LRR |
| 948503 | AT5G45240.1 | 8 | 2335572 | 2339307 | TIR-NBS-LRR |
| 356517 | AT5G45230.1 | 8 | 2347535 | 2350949 | TIR-NBS-LRR |
| 915611 | AT5G45210.1 | 8 | 2352151 | 2355810 | TIR-NBS-LRR |
| 915612 | AT5G45200.1 | 8 | 2362015 | 2369264 | TIR-NBS-LRR |
| 948510 | AT5G45220.1 | 8 | 2375814 | 2378129 | TIR-NBS-LRR |
| 356522 | AT5G45050.1 | 8 | 2379190 | 2380372 | TIR-NBS-LRR |
| 915616 | AT5G45220.1 | 8 | 2384891 | 2385889 | TIR-NBS-LRR |
| 948513 | AT4G36140.1 | 8 | 2388152 | 2390593 | TIR-NBS-LRR |
| 948514 | AT4G36150.1 | 8 | 2395747 | 2400426 | TIR-NBS-LRR |
| 915619 | AT4G36140.1 | 8 | 2401105 | 2402572 | TIR-NBS-LRR |
| 915620 | AT4G36150.1 | 8 | 2404097 | 2410068 | TIR-NBS-LRR |
| 915648 | AT5G45050.1 | 8 | 2623905 | 2628796 | TIR-NBS-LRR |
| 494475 | AT5G45060.1 | 8 | 2646266 | 2650262 | TIR-NBS-LRR |
| 915658 | AT5G45050.1 | 8 | 2725209 | 2729313 | TIR-NBS-LRR |
| 915659 | AT5G45060.1 | 8 | 2736449 | 2740577 | TIR-NBS-LRR |
| 948566 | AT5G45060.1 | 8 | 2762445 | 2766432 | TIR-NBS-LRR |
| 915663 | AT5G45050.1 | 8 | 2768079 | 2773021 | TIR-NBS-LRR |
| 356572 | AT5G45000.1 | 8 | 2783312 | 2785111 | TIR-NBS-LRR |
| 915685 | AT5G44870.1 | 8 | 2875768 | 2878612 | TIR-NBS-LRR |
| 330904 | AT5G44870.1 | 8 | 2900324 | 2904200 | TIR-NBS-LRR |
| 915860 | AT1G51480.1 | 8 | 4013005 | 4015598 | CC-NBS-LRR |
| 915866 | AT5G43740.2 | 8 | 4065680 | 4068242 | CC-NBS-LRR |
| 948891 | AT5G45220.1 | 8 | 4848450 | 4849909 | TIR-NBS-LRR |
| 915999 | AT5G11250.1 | 8 | 5469577 | 5471052 | TIR-NBS-LRR |
| 916001 | AT5G11250.1 | 8 | 5490035 | 5505248 | TIR-NBS-LRR |
| 916055 | AT5G22690.1 | 8 | 5996454 | 5999712 | TIR-NBS-LRR |
| 916056 | AT5G46470.1 | 8 | 6006641 | 6008110 | TIR-NBS-LRR |
| 356864 | AT5G63020.1 | 8 | 6072608 | 6073457 | CC-NBS-LRR |
| 949011 | AT5G63020.1 | 8 | 6336648 | 6337048 | CC-NBS-LRR |
| 916368 | AT5G43470.2 | 8 | 11959557 | 11961494 | CC-NBS-LRR |
| 916369 | AT5G48620.1 | 8 | 11967569 | 11969207 | CC-NBS-LRR |
| 494924 | AT5G43470.1 | 8 | 11976430 | 11982186 | CC-NBS-LRR |
| 951539 | AT5G66910.1 | 8 | 22541584 | 22541790 | CC-NBS-LRR |
| 951540 | AT5G66910.1 | 8 | 22542073 | 22544623 | CC-NBS-LRR |
| 496910 | AT5G66900.1 | 8 | 22545035 | 22547851 | CC-NBS-LRR |

**Table S6: *Arabidopsis thaliana* NLR resistance genes with *a priori* evidence for balancing selection**. These loci (as defined by their TAIR IDs) show evidence in independent studies for being influenced by balancing selection, but also show analogs in *Arabidopsis lyrata* that are located within high diversity genomic regions in one or more sample groups in the present study. Where possible, we have provided a published gene name and pathogen group (oomycete, fungal, bacterial) against which this gene has been demonstrated to provide disease resistance in *A.* *thaliana* (reference number in square brackets and listed below). Evidence for balancing selection in the gene in different species in independent studies are summarised here as: high diversity (nucleotide diversity, high numbers of alleles/protein variants), high Ka/Ks (evidence for elevated substitution rates at non-synonymous sites, Ka, relative to synonymous sites, Ks, suggesting positive selection), long evolutionary history (long-term maintenance and divergence of alleles across diverged lineages within or between species), reduced genetic differentiation (as estimated by F_ST_ among different populations), elevated Tajima’s D (indicating intermediate allele frequencies within sample groups) and shared polymorphism (among populations/related species), along with the corresponding reference (in square brackets) and species used in the study. The numbered references refer to: [1] Warren et al (1998); [2] Borhan et al (2008); [3] Holub (2007); [4] Staal et al (2006); [5] Grant et al (1995); [6] Botella et al (1998); [7] Bittner-Eddy et al (2000); [8] Lewis et al (2010); [9] Parker et al (1997); [10] Bent et al (1994); [11] McDowell et al (1998); [12] Tian et al. (2002); [13] Karasov et al. (2014); [14] Gos et al. (2012); [15] Bakker et al. (2006); [16] Cork & Purugganan (2005); [17] Stahl et al. (1999); [18] Bergelson et al. (2001); [19] Rose et al. (2004); [20] Ding et al. (2007); [21] Noel et al. (1999); [22] Sicard et al. (2015); [23] Mauricio et al. (2003).

| **AIR Gene ID** | ***R-*gene name (type)** | **Presence in high diversity regions** | **Evidence for balancing selection [reference]** | **Species** |
| --- | --- | --- | --- | --- |
| AT1G12220 | *RPS5* (bacterial) [1] | NA-S | Long evolutionary history [12] | *A. thaliana* |
|  |  |  | High diversity; long evolutionary history, elevated Tajima’s D [13] | *A. thaliana* |
| AT1G56540 | *wrr4* paralog (oomycete) [2] | EU-N, NA-O, ALL-O | High diversity, reduced genetic differentiation, shared polymorphism [14] | *C. grandiflora* |
|  |  |  | High diversity, reduced genetic differentiation, shared polymorphism [14] | *C. rubella* |
|  |  |  | High diversity; high Ka/Ks, elevated Tajima’s D [15] | *A. thaliana* |
| AT1G59780 | *rpp7* cluster (oomycete) [3] | NA-S, EU-N, NA-O, ALL-O | High diversity; high Ka/Ks, elevated Tajima’s D [15] | *A. thaliana* |
| AT1G63730 | No attributed function | EU-N, NA-O, ALL-O | High diversity, reduced genetic differentiation, shared polymorphism [14] | *C. rubella* |
| AT1G63870 | *rlm1B* homolog (fungal)[4] | EU-N, NA-O, ALL-O | High diversity [16] | *A. thaliana* |
| AT1G63880 | *RLM1B* (fungal) [4] | Not present | High diversity [16] | *A. thaliana* |
| AT3G07040 | *RPM1* (bacterial) [5] | NA-O | High diversity; long evolutionary history [17] | *A. thaliana* |
|  |  |  | Long evolutionary history [18] | *A. thaliana* |
| AT3G44480 | *RPP1* (oomycete) [6] | NA-S, EU-N, NA-O, ALL-O | Long evolutionary history [18] | *A. thaliana* |
| AT3G46530 | *RPP13* (oomycete) [7] | EU-N, NA-O, ALL-O | High diversity, high Ka/Ks, elevated Tajima’s D [15] | *A. thaliana* |
|  |  |  | High diversity [19] | *A. thaliana* |
|  |  |  | High diversity [20] | *A. thaliana* |
|  |  |  | Long evolutionary history [18] | *A. thaliana* |
| AT3G50950 | *ZAR1* (bacterial) [8] | Not present | High diversity; high Ka/Ks, elevated Tajima’s D [15] | *A. thaliana* |
| AT4G14370 | No attributed function | Not present | High diversity; high Ka/Ks, elevated Tajima’s D [15] | *A. thaliana* |
| AT4G14610 | No attributed function | Not present | High diversity; high Ka/Ks, elevated Tajima’s D [15] | *A. thaliana* |
| AT4G16950 | *RPP5* (oomycete) [9] | NA-S | High diversity [21] | *A. thaliana* |
|  |  |  | Shared polymorphism [22] | *Capsella* |
| AT4G26090 | *RPS2* (bacterial) [10] | NA-S, NA-O | High diversity [20] | *A. thaliana* |
|  |  |  | Long evolutionary history [18, 23] | *A. thaliana* |
| AT5G43470 | *RPP8* (oomycete) [11] | EU-N | High diversity [20] | *A. thaliana* |
|  |  |  | Long evolutionary history [18] | *A. thaliana* |
|  |  |  | High diversity, high Ka/Ks [12] | *A. thaliana/A.lyrata* |
| AT5G58120 | No attributed function | Not present | High diversity; high Ka/Ks, elevated Tajima’s D [15] | *A. thaliana* |
| AT5G63020 | No attributed function | NA-S, EU-N | High diversity; high Ka/Ks, elevated Tajima’s D [15] | *A. thaliana* |

**Table S7:** ***A. lyrata* genes with multiple lines of evidence for balancing or diversifyling selection in our study.** *Arabidopsis lyrata* NLR analogs (and also several NB-ARC and TIR-class) identified within genomic regions of elevated diversity in our study that also show additional signatures of balancing selection (reduced F_ST_ and/or elevated Taijma’s D) or diversifying selection (elevated F_ST_). The TAIR ID and name for a defense-related gene in *A.thaliana*, along with the *A. lyrata* locus name (phytozome 107) is given. Five of these TAIR genes also show signatures of balancing selection in independent studies (highlighted in bold with ** next to TAIR gene ID) as described in Table S6. The seminal reference is provided for data describing a functional allele in *A. thaliana* and pathogen group (oomycete, fungal, bacterial) against which this gene has been demonstrated to provide disease resistance in *A.* *thaliana* (reference number in square brackets and listed below). The final two columns describe the presence of the corresponding *A. lyrata* locus in genomic regions of elevated diversity for different sample groups (NA-S, NA-O, EU-N or ALL-O), together with any additional evidence for balancing selection acting in this genomic region for different sample groups. The references associated with this table are: [1] Borhan *et al.* 2008, [2] Holub 2007, [3] Staal *et al.* 2006, [4] Sepahvand and Holub, unpublished, [5] Botella *et al.* 1998, [6] Lolle *et al.* 2017, [7] Sinapidou *et al.* 2004, [8] Sarazin *et al.* 2015, [9] McDowell *et al.* 1998, [10] Lee *et al.* 1996, [11] Saucet *et al.* 2015, [12] Gassman *et al.* 1999, [13] Deslandes *et al.* 2004

| **TAIR Gene ID** | ***A. lyrata* locus name(s)** | ***R-*gene name (resistance type) [reference]** | **Presence in high diversity regions** | **Additional evidence for selection** |
| --- | --- | --- | --- | --- |
| AT1G15890 | 937702 | None found | All-OUT | High F_ST_ |
|  | 920497 |  | EU-N | High D (Iceland) |
| AT1G27180 | 930688 | None found | All-OUT, EU-N | High F_ST_, High D (Norway, Iceland) |
| AT1G50180 | 474150 | None found | NA-O | High F_ST_, High D |
| ****AT1G56540** | **914939, 914947** | ***WRR4* paralog (oomycete) [1]** | **NA-O** | **High D** |
|  | **924354** |  | **EU-N** | **High D (Norway)** |
| **AT1G58390** | **914885** | ***RPP7* cluster (oomycete) [2]** | **EU-N** | **High D (Norway)** |
| **AT1G59620** | **475351** | ***RPP7* cluster (oomycete) [2]** | **NA-O** | **High D** |
| ****AT1G59780** | **907538** | ***RPP7* cluster (oomycete) [2]** | **NA-O** | **High D** |
|  | **891414** |  | **EU-N** | **High D (Iceland)** |
| **AT1G63730 | 907022, 907036 | None found | EU-N | High F_ST_ |
| AT1G63740 | 474976 | None found | EU-N | High F_ST_ |
| **AT1G63870 | 337990, 907024 | *RLM-B* homolog (fungal) [3] | EU-N | High F_ST_ |
| AT1G65850 | 908051 | None found | All-OUT | High F_ST_ |
|  | 893040 |  | NA-O | High D |
| AT2G14080 | 895001 | *RPP28* (oomycete) [4] | NA-O | Low F_ST_ |
| AT2G16870 | 914883 | None found | EU-N | High D (Norway) |
| AT3G25510 | 891298 | None found | All-OUT | High F_ST_ |
| **AT3G44400** | **915002, 915006** | ***RPP1* cluster (oomycete) [5]** | **All-OUT, NA-O** | **High F_ST_ (All-O), High D (NA-O)** |
| **AT3G44630** | **895002** | ***RPP1* cluster (oomycete) [5]** | **NA-O** | **Low F_ST_** |
| AT4G11340 | 895306 | None found | NA-O | High F_ST_ |
| AT4G12010 | 327329 | *DSC1* [6] | EU-N | High F_ST_ |
| AT4G19510 | 915589, 890225 | *RPP2-B* (oomycete) [7] | NA-O | High D |
|  | 915589 |  | EU-N | High D (Norway) |
| AT4G19520 | 915597, 946227 | *RPP2-A* (oomycete) [7] | NA-O | High D |
| AT4G23510 | 889815 | None found | NA-O | High D |
| AT4G36150 | 932480, 320248 | None found | All-OUT | High F_ST_ |
| AT5G11250 | 915999, 916001 | *BNT1* (general stress response) [8] | EU-N | High D (Norway, Sweden) |
| AT5G38340 | 891930 | None found | All-OUT, EU-N | High F_ST_ (All-O), High D (Sweden) |
| AT5G38350 | 891927, 891933 | None found | All-OUT, EU-N | High F_ST_ (All-O), High D (Sweden, Iceland) |
| AT5G38850 | 891858 | None found | EU-N | High D (Norway, Sweden, Iceland) |
| AT5G40090 | 493978 | None found | All-OUT | High F_ST_ |
| AT5G40100 | 947767 | None found | All-OUT | High F_ST_ |
| ****AT5G43470** | **494924, 916368** | ***RPP8* (oomycete) [9]** | **EU-N** | **High F_ST_ (EU-N), High D (Iceland)** |
| AT5G44870 | 915685 | *LAZ5, TTR1* [10] | NA-O | High D |
| AT5G45000 | 356572 | None found | NA-O | High D |
| AT5G45050 | 915594, 915658, 915663, 948494 | *RRS1B* [11] | NA-O | High D |
| AT5G45060 | 915593, 915659, 948495, 948566 | *RPS4B* [11] | NA-O | High D |
| AT5G45220 | 948891 | *RPS4/RRS1* cluster [12,13] | All-OUT | High Fst |
| AT5G45230 | 915600 | *RPS4/RRS1* cluster [12,13] | NA-O | High D |
| AT5G45240 | 948480, 948493, 948499 | *RPS4/RRS1* cluster [12,13] | NA-O | High D |
|  | 948480 |  | EU-N | High D (Norway) |
| AT5G45250 | 915595, 915598, 948472 | *RPS4* (bacterial) [12] | NA-O | High D |
|  | 948472 |  | EU-N | High D (Norway) |
| AT5G45260 | 915586, 915590, 948486 | *RRS1* (bacterial) [13] | NA-O | High D |
|  | 915586, 915590 |  | EU-N | High D (Norway) |
| AT5G48620 | 916369 | None found | EU-N | High F_ST_, High D (Iceland) |

**FIGURES**

**Figure S1**. **Number of reads per individual for European and North American *Arabidopsis lyrata.*** (a) The total number of reads; (b) the number of reads aligning once to the reference; (c) the number of reads aligning more than once to the reference; (d) the number of reads that did not align. The significance of the difference in median read numbers (horizontal black line on box plots) was tested using 1000 permutations of the country of origin category among individuals ; the *p*-value for the difference is given above each box plot.

**Figure S2**: **Genetic clustering of samples across subspecies and within Europe**. Genetic structure as described by PCA (principal component axis 1 and 2) among (a) all 91 samples of *A. lyrata* using 5941 polymorphic loci*;* and (b) all 37 Northern European samples using 4580 polymorphic loci. The amount of variance explained by each principal component is given on the axis labels.

**Figure S3**: **Isolation-by-distance (IBD) within Northern European countries and the Great Lakes region.** IBD relationships as estimated using F_ST_/(1-F_ST_) plotted against geographic distance (km) among populations within: (a) Norway; (b) Sweden; (c) Iceland; and (d) all North American outcrossing samples. The significance of regressions of geographic distance on genetic distance are given above each graph (as estimated using the Multiple Regression on Matrices function in the “ecodist” R package). Solid regression lines indicate a significant relationship (p<0.05) and dashed indicate non-significant relationships (p>0.05).

**Figure S4**: **Genome-wide patterns of coverage average within sample groups with or without missing data**. (a-d) Coverage estimated for those loci present at a minimum of 10 x coverage in all individuals (5942 loci) and (e-h) loci present in at least 50% of individuals for each group (16240 loci). The horizontal line represents the mean coverage for each regional group. The shading denotes the eight chromosomes of *A. lyrat*a; position along the reference genome is given in Mega base pairs.

**Figure S5**. **Variation in individual observed heterozygosity (*H_o_*) across RAD loci with up to 50% missing data**. The proportion of 17936 RAD loci present in at least 50% of the individuals that are heterozygous for (a) the 42 individuals sampled from Europe; and (b) the 49 individuals sampled from the North American Great Lakes (outcrossing = dark grey, Great Lakes inbreeding = white). The gaps between sets of bars indicate different populations within countries (a) or different populations across the Great Lakes region (b).

**Figure S6. Differences in the density of NLR genes between genomic regions of elevated and non-elevated polymorphism.** Histograms showing the permuted difference (1000 permutations) in average density of NLR genes, in smoothed windows of significantly elevated diversity relative to all other windows. The arrow indicated the observed difference and the p-value is given (proportion of times the permuted difference was greater than observed). In all but one graph RAD loci present in at least 50% individuals in a sample group (<50% missing) were used. a) N. American selfing (0% missing data), b) N. American selfing (>50% missing data), c) Northern European, d) N. American outcrossing, and e) all outcrossing samples.

**Figure S7**: **Differences in the density of LRR-RLK genes between genomic regions of elevated and non-elevated polymorphism** Histograms showing the permuted difference (1000 permutations) in average density of LRR-RLK genes, in smoothed windows of significantly elevated diversity relative to all other windows. The arrow indicated the observed difference and the p-value is given (proportion of times the permuted difference was greater than observed. In all but one graph RAD loci present in at least 50% individuals in a sample group (<50% missing) were used. a) N. American selfing (0% missing), b) N. American selfing (<50% missing), c) Northern European, d) N. American outcrossing, and e) all outcrossing samples.

**Figure S8. Genomic regions of significantly elevated polymorphism across North American and European outcrossing samples.** Smoothed nucleotide diversity across loci present in at least 50% of (a) all EU-N samples (N=35) and (b) all NA-O samples (N=28). Genomic regions (formed by merging nieghbouring smoothed windows) of significantly elevated nucleotide and/or gene diversity are marked in purple. The eight chromosomes of *A. lyrata* are shaded alternately in dark and light grey. The location of 206 genes annotated as “disease resistance” on the *A. lyrata* reference chromosomes 1-8 are denoted by blue squares. Interesting candidate loci showing independent signatures of balancing selection in related species (see Table S6) are marked with blue arrows (with likely gene clusters indicated by brackets containing the number of neighbouring *A. lyrata* loci annotated as this NLR gene).

**Figure S9**: **Distribution of Tajima’s D values for overlapping 120 kbp windows across samples within geographic regions**. D estimated across all samples in (a) the North American outcrossing group (NA-O, N=32); (b) Norway (EU-N, N=11), c) Sweden (EU-N, N=12) and d) Iceland (EU-N, N=12). Tajima’s D was estimated the R package “PopGenome”, and windows were incrementally moved long the genome by 12 kbp each time.

**Figure S10**: **Patterns of genetic differentiation and Tajima’s D in sliding genomic windows across the genome for EU-N samples**. (a) Smoothed genetic differentiation among Norway, Sweden and Iceland (EU-N) estimated using loci present in at least 50% of individuals within each country. (b) 120Kbp sliding window estimates of Tajima’s D for, Norway (N=11). Similar plots are given for (c) Sweden and (d) Iceland. (a) 120 kbp sliding window estimates of Tajima’s D for Sweden (N=12), and (b) Iceland samples (N=12). Windows showing elevated (green) or reduced (magenta) F_ST_ in a region of elevated diversity are indicated in (a), with regions of elevated Tajima’s D in regions of significantly elevated diversity (green) indicated in (b-d). The location of 206 genes annotated as “disease_resistance” on the *A. lyrata* reference chromosomes 1-8 are denoted by blue squares, with those in the windows overlapping with regions of elevated diversity highlighted in green.

**Figure S11**: **Patterns of genetic polymorphism and genetic differentiation in sliding genomic windows for all outcrossing samples.** (a) Smoothed nucleotide diversity across 16990 loci present in at least 50% of all outcrossing samples from Northern Europe and N. America (N=72), and (b) genome-wide differentiation between the European and North American subspecies, using loci present in at least 50% of individuals in each subspecies. Regions of significantly elevated nucleotide and/or gene diversity are marked in vertical purple lines and windows showing elevated (green) or reduced F_ST_ (magenta) in a region of elevated diversity are indicated. Interesting candidate loci showing independent signatures of balancing selection in related species (see Table S6) are marked with blue arrows (with likely gene clusters indicated by brackets containing the number of neighbouring *A. lyrata* loci annotated as this NLR gene).

**Figure S12**: **The effect of varying individual locus coverage on SNP calling and locus retention.** The effects of varying the number of reads allowed per locus on (a) the proportion uncalled nucleotide sites (i.e. those that could not be called confidently as heterozygous or homozygous); (b) the proportion of heterozygous sites; and (c) the proportion of reads retained per individual. Box plots give the median, the first and third quantile and the 95% confidence interval of median for all 91 individuals. The proportion of uncalled and heterozygous sites are given after SNP calling corrections conducted by the rxstacks module.
